# Dehydration prompts increased activity and blood feeding by mosquitoes

**DOI:** 10.1038/s41598-018-24893-z

**Published:** 2018-05-01

**Authors:** Richard W. Hagan, Elise M. Didion, Andrew E. Rosselot, Christopher J. Holmes, Samantha C. Siler, Andrew J. Rosendale, Jacob M. Hendershot, Kiaira S. B. Elliot, Emily C. Jennings, Gabriela A. Nine, Paula L. Perez, Alexandre E. Rizlallah, Miki Watanabe, Lindsey E. Romick-Rosendale, Yanyu Xiao, Jason L. Rasgon, Joshua B. Benoit

**Affiliations:** 1 Department of Biological Sciences, University of Cincinnati, Cincinnati, OH 45221 USA; 2 Department of Mathematical Sciences, University of Cincinnati, Cincinnati, OH 45221 USA; 3 Division of Pathology, Cincinnati Children’s Hospital Medical Center, Cincinnati, OH 45229 USA; 4 Department of Entomology, Center for Infectious Disease Dynamics and Huck Institutes for Life Sciences, Pennsylvania State University, University Park, Pennsylvania, PA 16802 USA

## Abstract

Current insights into the mosquito dehydration response rely on studies that examine specific responses but ultimately fail to provide an encompassing view of mosquito biology. Here, we examined underlying changes in the biology of mosquitoes associated with dehydration. Specifically, we show that dehydration increases blood feeding in the northern house mosquito, *Culex pipiens*, which was the result of both higher activity and a greater tendency to land on a host. Similar observations were noted for *Aedes aegypti* and *Anopheles quadrimaculatus*. RNA-seq and metabolome analyses in *C*. *pipiens* following dehydration revealed that factors associated with carbohydrate metabolism are altered, specifically the breakdown of trehalose. Suppression of trehalose breakdown in *C*. *pipiens* by RNA interference reduced phenotypes associated with lower hydration levels. Lastly, mesocosm studies for *C*. *pipiens* confirmed that dehydrated mosquitoes were more likely to host feed under ecologically relevant conditions. Disease modeling indicates dehydration bouts will likely enhance viral transmission. This dehydration-induced increase in blood feeding is therefore likely to occur regularly and intensify during periods when availability of water is low.

## Introduction

Dehydration represents a significant factor that impacts the geographical distribution, reproductive capacity, and longevity of terrestrial arthropods^1–4^. With more, and extended, periods of drought predicted to occur as climate change progresses^5,6^, the ability to tolerate bouts of dehydration is becoming increasingly critical to insect survival. Water loss for terrestrial invertebrates occurs through excretion, respiratory gas exchange, and cuticle transpiration^7,8^. Insects residing in dry environments significantly minimize water loss through mechanisms that suppress rates of cuticular water loss, reduce excretion, and behaviorally by retreating into more favorable microhabitats^8,9^. If water balance cannot be maintained, a complement of mechanisms is employed to prevent damage from osmotic stress. These changes include increased expression of heat shock proteins^3,8,10,11^, shifts in osmoprotectant metabolites^10–13^, and differential regulation of aquaporins to alter the movement of water between the hemolymph and intracellular components^14–16^. Along with these physiological responses, dehydration will prompt moisture-seeking behavior in many insects as a means to increase the likelihood of liquid ingestion and reduce water loss^3,17,18^.

Dehydration resistance in mosquitoes is a significant factor in determining their ability to expand to new regions and also dictates their survival under periods of low water availability^7,19–23^. For mosquitoes that act as malaria vectors, recent studies have examined molecular and physiological changes associated with dehydration or dry-season conditions^22–24^. Rearing *Anopheles gambiae* under dry season conditions shifted levels of specific metabolites (amino acids and carbohydrates), suppressed reproduction, reduced spiracle size, and reduced heart contraction rate^21–27^. Microarray analyses of dehydrated *A*. *gambiae* revealed increased expression of genes associated with stress response and DNA repair^24^. Rehydration in mosquitoes can be achieved through three main routes: ingestion of free water, nectar feeding, and hematophagy^19^. For select mosquito species, specifically two of the most important disease vectors, *A*. *gambiae* and *A*. *aegytpi*, individuals will forgo nectar feeding and imbibe multiple bloodmeals during each gonotrophic cycle^28–30^. Importantly, these approaches to nectar or blood feeding can vary greatly between species and strains of mosquitoes^28–30^. However, even species that have a preference for blood feeding will ingest nectar^31^, suggesting the potential for rehydration through sugar feeding for all species. Although previous studies have identified many interesting aspects associated with the dynamics between dehydration and mosquito physiology, none have examined the effects of dehydration bouts on mosquito biology using integrative studies that range from behavior to utilization of RNA interference techniques to assess specific phenotypic changes.

In this study, we examined the effect of hydration status on the physiology and behavior of mosquitoes via RNA-seq analyses along with blood feeding, host landing, and physiological assays. Our hypothesis is that dehydration will alter metabolism, shift activity, and increase levels of blood feeding for the northern house mosquito, *Culex pipiens*. We found that short periods of dehydration prompted an increase in transcript levels of genes associated with carbohydrate catabolism, increased activity, promoted mosquito host landing, and elevated blood feeding. Offering a source of drinking water under dry conditions or rehydration following dehydration suppressed these phenotypic changes, suggesting that dehydration, rather than exposure to low relative humidity (RH), prompts the observed phenotypes. Similar results for select experiments were noted for two other mosquito species, *Aedes aegypti*, and *Anopheles quadrimaculatus*, suggesting that these findings are generalizable across mosquito species in both the Culicinae and Anophelinae subfamilies. Mesocosm-based studies indicated that hydration status impacts blood feeding phenotypes under field conditions even if free water sources are available. Furthermore, modeling of our findings provide support to the hypothesis that increased blood feeding, due to dehydration, will potentially increase disease transmission. Our study indicates that bouts of dehydration, either induced by dry conditions, mosquitoes not ingesting nectar or water, or a combination of both, alters the physiology and subsequent behavior of mosquitoes in a manner that has significant implications for mosquito-environment-blood feeding dynamics.

## Results

### Shifts in activity and blood feeding after dehydration stress

**Figure 1:**
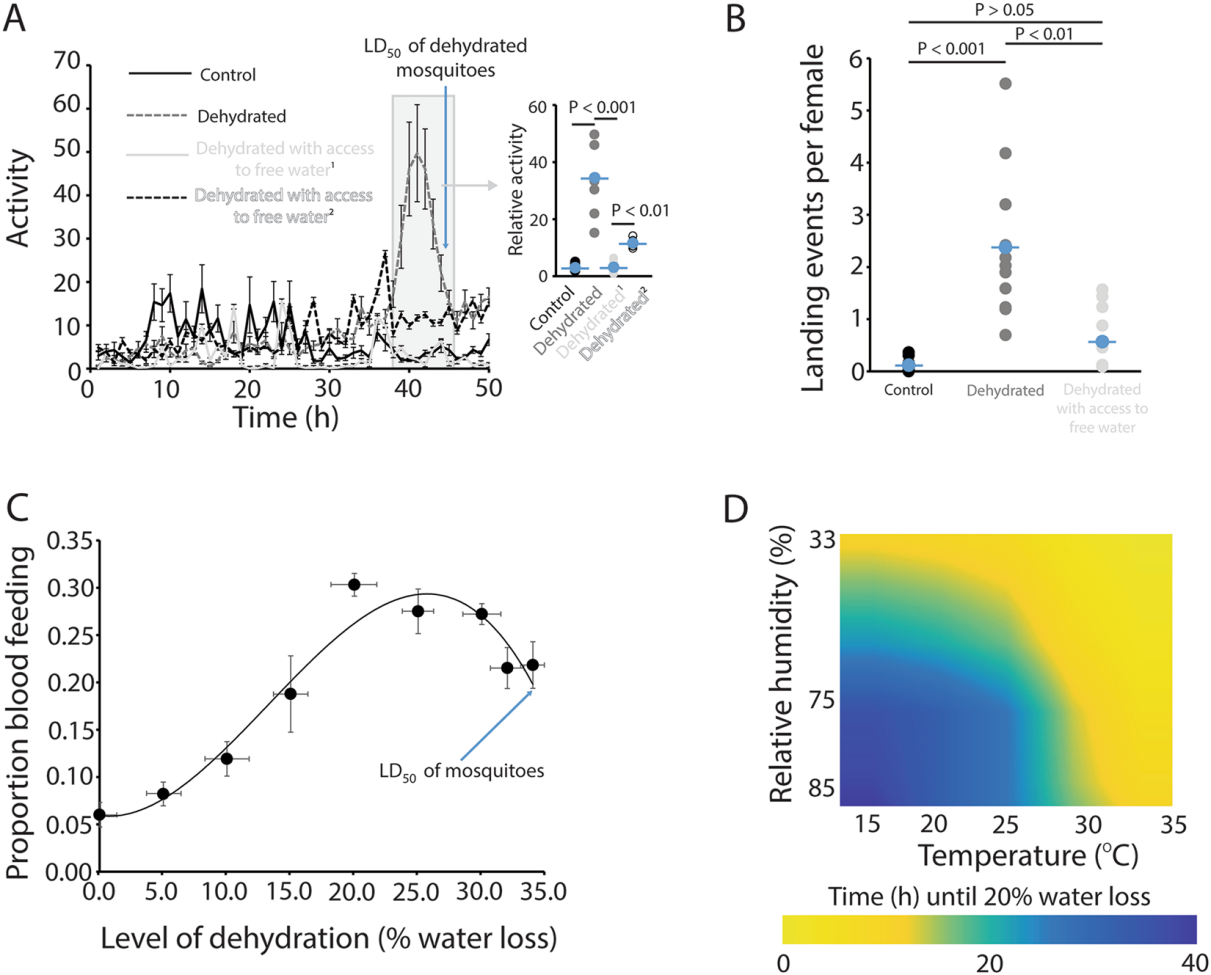
Dehydration increased mosquito activity and blood feeding for *C*. *pipiens* females. (**A**) Activity measured by a Locomotor Activity Monitor 25 (LAM25) system through the course of dehydration, inset represents averaged results from 38–44 hours. Each point is the mean ± SE of 36–48 mosquitoes. ^1^Mosquitoes have access to water for drinking to remain hydrated. ^2^Free water is present, but mosquitoes are prevented from drinking by a mesh barrier. (**B**) Number of landing events per mosquito over the course of one hour. Mean ± SE represents 12 independent replicates of 30–40 mosquitoes. (**C**) Proportion of mosquitoes following varying levels of dehydration that blood fed within two hours of host availability. Mean ± SE represents 10 replicates of 50 mosquitoes. (**D**) Time until the 20% dehydration point under varying temperatures and relative humidity (RH). Three replicates of 40 mosquitoes were conducted at each RH (33, 75, and 100%) at each temperature. Statistical analyses for (**A**–**D**) were conducted by a t-test or one- and two-way ANOVA followed by Tukey’s HSD post-hoc analysis where appropriate.

During initial dehydration experiments, we observed that desiccated *C*. *pipiens* females appeared more active compared to control, non-dehydrated counterparts. Total activity for *C*. *pipiens* females was assessed under dehydrating (72–78% RH, referred to as 75% RH) and non-dehydrating (96–100% RH, referred to as 100% RH, or 75% RH with access to free water) conditions. Exposure to dehydration significantly increased activity after 28–30 hours (~15–20% water loss, Fig. S1), and activity levels were maintained until dehydration-induced mortality occurred at a loss of ~35% water content (Fig. 1A). An additional control where water was present at 75% RH, but mosquitoes were prevented from drinking, showed that resting near the water source only delayed dehydration. Importantly, water content of mosquitoes held at 100% RH and 75% RH with access to free water were similar to those collected from the colony and previous studies^8^, suggesting that hyper-hydration does not under occur under our treatments (Fig. S2). This indicates that retreat to a high RH refuge will only delay and likely extend dehydration-induced phenotypes over a longer timeframe (Fig. S2). Thus, significantly increased activity will occur for ~12–15 hours, or even longer if relative humidity is slightly higher, prior to dehydration-induced mortality.

In conjunction with increased activity, the propensity of mosquitoes to land on a host mimic increased nearly two-fold at a loss of ~10% water content and 4- to 5-fold after losing ~15–20% of water content at 75% RH, but not when held under non-dehydrating conditions (100% RH) or at 75% RH with access to liquid water for ingestion (Fig. 1B). This indicates that dehydration stress, not dry conditions, is responsible for the observed phenotypes. Comparative studies using *A*. *aegypti* and *A*. *quadrimaculatus*, confirmed that dehydration-induced increases in both activity and host landing are likely a general response across diverse mosquito species (Fig. S1–S3). Lastly, the propensity of *C*. *pipiens* to ingest a bloodmeal increased significantly after a 10–15% decrease in water content (~15% of female feeding) and was the highest when water loss was 20–30% (~30% of females feeding; Fig. 1C). The baseline proportion of mosquitoes that would blood feed was ~5% before dehydration stress and remained at this level when mosquitoes were held at hydrating conditions.

When temperature and relative humidity were examined in relation to dehydration levels, the dehydration threshold of 10% and 20% water loss for *C*. *pipiens* females can be reached within as little as three to four or six to eight hours, respectively, at low (Fig. 1D), but ecologically relevant, RHs (60–80%) and summer temperatures (26–28 °C) common within the Midwestern United States. These results indicate that dehydration bouts increase the propensity of females to ingest a bloodmeal and this impact can be exacerbated by temperature and RH dynamics that will occur under ecologically relevant conditions.

### Transcriptional and metabolomic changes associated with *C. pipiens* dehydration

**Figure 2:**
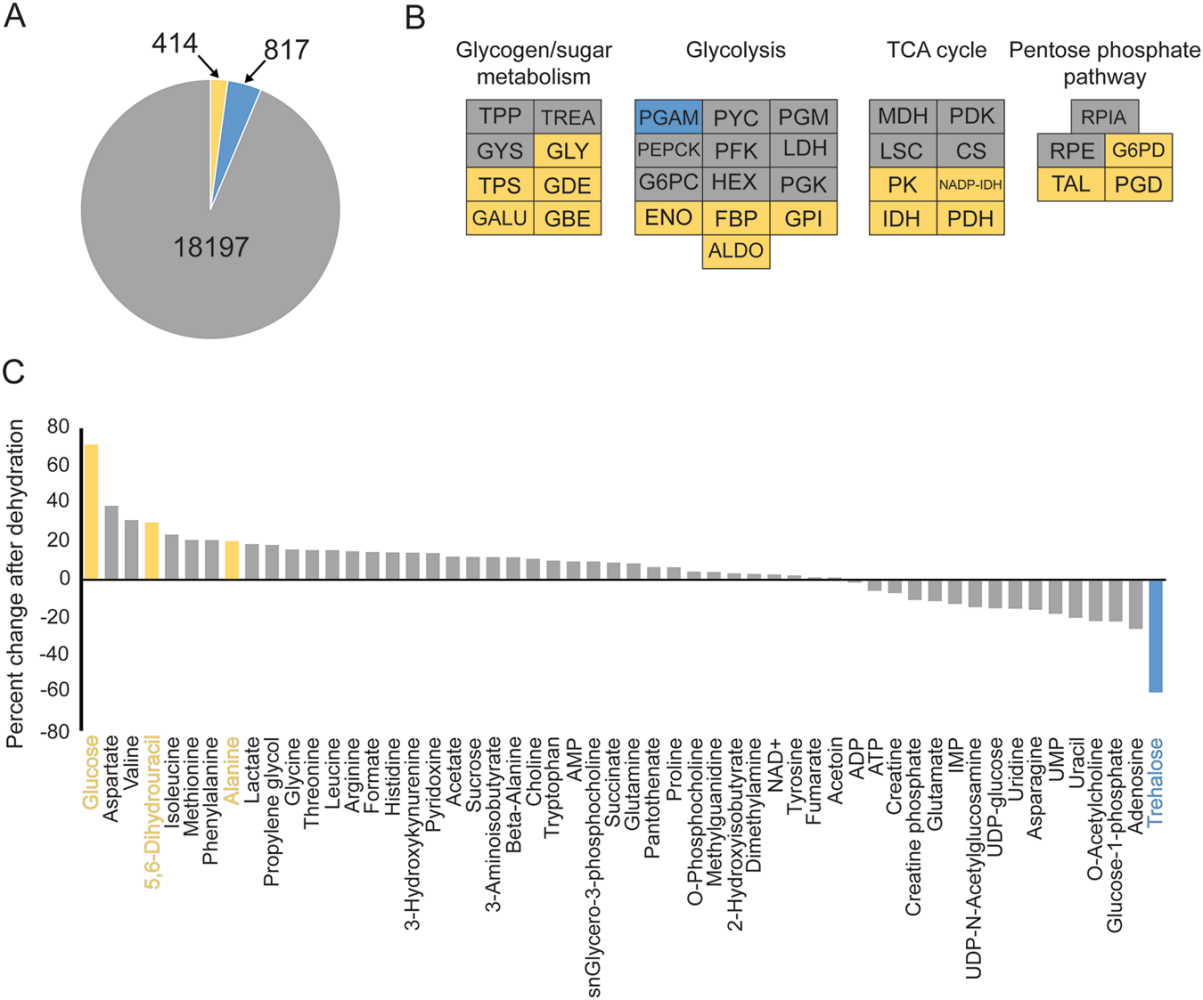
Dehydration enriched carbohydrate metabolism and prompted a trehalose to glucose shift for *C*. *pipiens* females. Blue denotes decreased levels during dehydration, gray indicates no difference, and yellow is increased during dehydration compared to hydrated individuals. (**A**) RNA-seq analyses following dehydration yielded differential expression of 1231 genes. (**B**) Expression levels for multiple carbohydrate-associated genes, many of which were upregulated. (**C**) Metabolomics following dehydration stress. Each value represents the average of eight replicates of combined samples with 20–30 mosquitoes.

To investigate the underlying mechanisms associated with the dehydration-induced phenotype, we utilized a combined RNA-seq and metabolomic approach in *C*. *pipiens* females. Dehydration exposure (15–20% loss of water) yielded over 1200 genes with differential transcript levels compared to fully hydrated individuals, a subset of which was validated through quantitative PCR (Fig. 2A, Fig. S4, Table S1). This gene set showed enrichment of Gene Ontology terms associated with carbohydrate metabolism (Fig. 2B, Table S2). Metabolomic analyses revealed a substantial increase in the levels of glucose during dehydration stress, supporting our RNA-seq data (Fig. 2C). Trehalose content declined by over 60% and is likely the source of the increase in glucose. Of interest, nearly half the glucose generated by the breakdown of trehalose is likely metabolized since the conversion noted here is only 1:1 rather than 2:1 as expected. These results indicate that altered carbohydrate metabolism, specifically the breakdown of trehalose to glucose, may underlie the phenotypic changes following dehydration in *C*. *pipiens*.

### Trehalose levels during dehydration and suppression of trehalose metabolism

**Figure 3:**
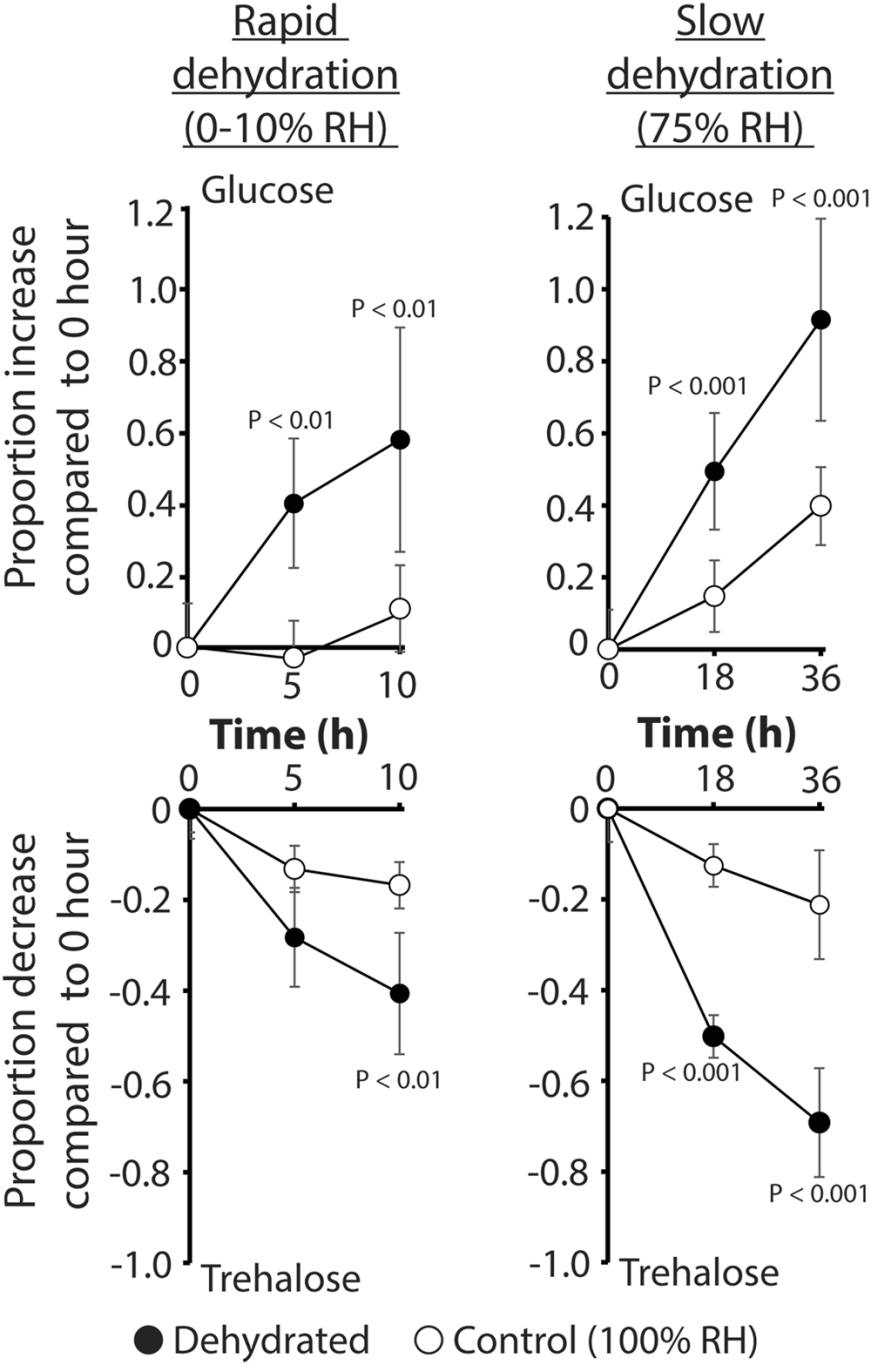
Glucose and trehalose changes are related to the rate of dehydration for *C*. *pipiens* females. Rapid and slow dehydration differentially altered levels of glucose and trehalose. Mean ± SE for 10 replicates of samples from four-five mosquitoes at each time point. Statistical values refer to the control versus dehydrated mosquitoes at each time point.

Previous studies indicated that slow dehydration induces more pronounced physiological and behavioral responses, when compared to rapid dehydration^11^. Similar to these metabolomic studies, glucose increased and trehalose decreased in *C*. *pipiens* females following dehydration exposure when measured with spectrophotometric assays (Fig. 3). The increase of glucose and decrease of trehalose was slightly augmented under slow dehydration (75% vs. 0% RH), suggesting that the rate of dehydration has a substantial impact on dehydration-induced phenotypes (Fig. 3). Glycogen levels in response to dehydration were examined since this reserve serves as a major source of trehalose (Fig. S5). We noted differences between control and dehydrated mosquitoes under slow dehydration (75% RH), which had a significant, albeit slight, decline in glycogen (Fig. S5). Mosquitoes held under dehydrating conditions with access to free water showed no reduction in glycogen levels compared to those that experienced dehydration stress without water (Fig. S5). Starvation is not likely serving as a major factor for our observed phenotypes in *C*. *pipiens* females since mosquitoes held at higher relative humidity, or allowed access to free water, survive for nearly four to five days before starvation-induced death (*p* < 0.0001, log-rank test; Fig. S6). These results suggest that a bout of dehydration alters trehalose and glycogen metabolism, thus generating increased levels of glucose. This phenotype is augmented when dehydration occurs at a slower rate.

**Figure 4:**
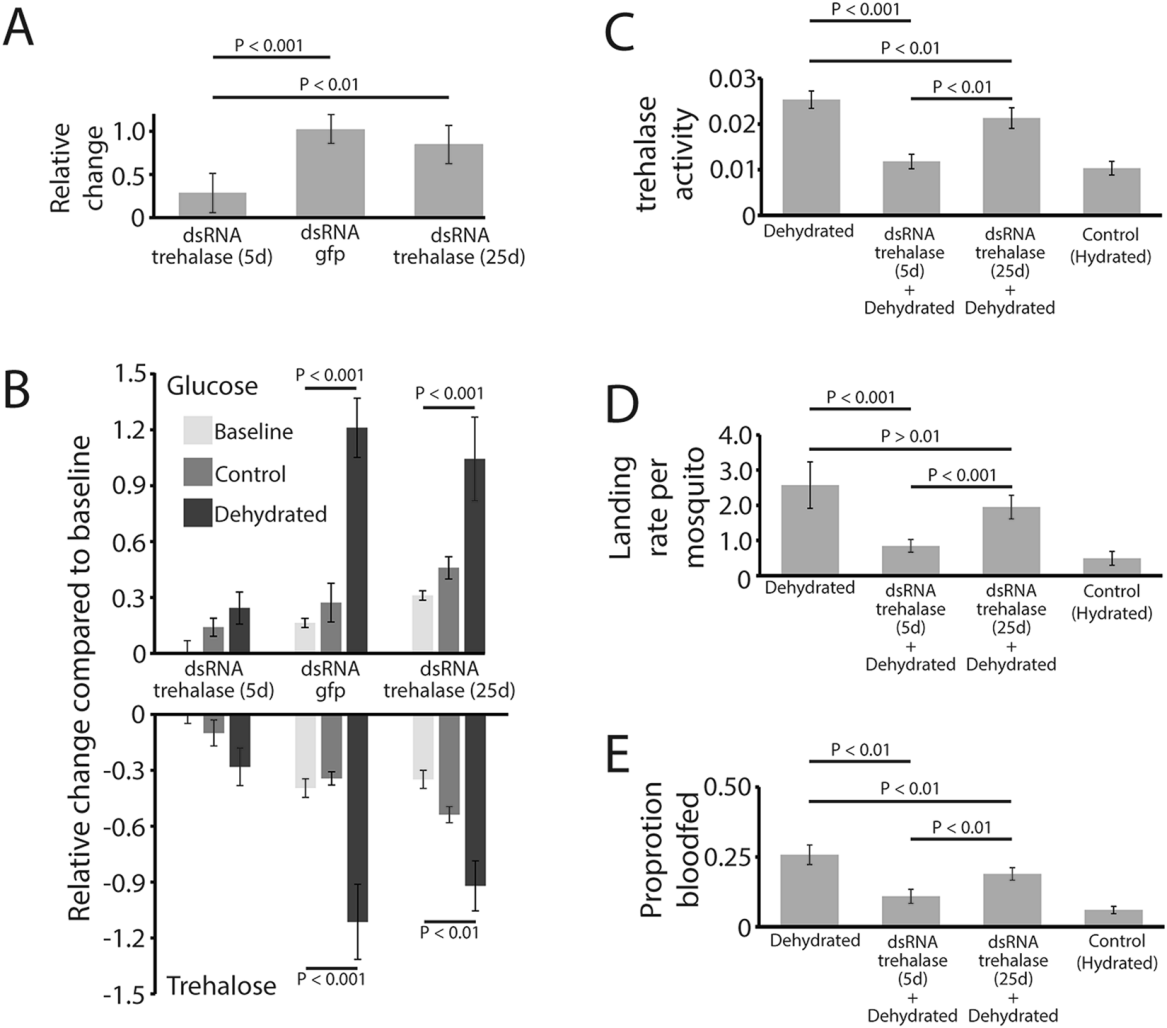
Glucose and trehalose shifts directly impacted mosquito blood feeding-dehydration dynamics for *C*. *pipiens* females. (**A**–**C**) Knockdown of *trehalase* prevented the trehalose to glucose conversion during dehydration by suppressing trehalase activity. Trehalase activity is in mol glucose/µg protein/minute. Mean ± SE for 12 mosquitoes at each time point. (**D**,**E**). Landing on the host (mean ± SE for 8 replicates at each time point) and blood feeding (mean ± SE for 8 replicates at each time point) changed following *trehalase* suppression and dehydration. Statistical analyses were conducted by a one- or two-way ANOVA followed by Tukey’s HSD post-hoc analysis where appropriate. 5d and 25d are periods following injection of dsRNA. Control, mosquitoes held at 100% RH for 18 h. Dehydrated, mosquitoes held at 75% RH for 18 h. Baseline denotes levels before 18 h of dehydration.

To link metabolism of trehalose with shifts in blood feeding, we utilized RNA interference to suppress the levels of *trehalase*, the enzyme involved in the conversion of trehalose to glucose, during the course of dehydration in *C*. *pipiens* female. dsRNA injection targeting *trehalase* in adult females resulted in nearly an 80% reduction in transcript levels and 53% lower trehalase activity compared to control mosquitoes (Fig. 4A–C). *trehalase* transcript levels recovered 25 days after dsRNA injection (Fig. 4A). When trehalose and glucose were measured following *trehalase* knockdown, there was a slight, non-significant trehalose decrease and glucose increase following dehydration stress (Fig. 4B). This lack of trehalose to glucose conversion after *trehalase* suppression was in stark contrast to shifts in trehalose and glucose observed when mosquitoes were injected with dsRNA targeting *green fluorescent protein* (*gfp*) or when the impact of dsRNA targeting *trehalase* dissipated after 25 days (Fig. 4B). The proportion of mosquitoes that landed on a host mimic following dehydration was reduced by over 50% after knockdown of *trehalase* and the response was partially recovered 25 days after dsRNA injection (Fig. 4D). Similarly, increased blood feeding in dehydrated mosquitoes was suppressed following knockdown of *trehalase* and recovered once the dsRNA effects began to decline after 25 days (Fig. 4E). Thus, the inhibition of trehalose to glucose conversion during dehydration inhibits the dehydration-induced phenotype of increased host interactions.

### Laboratory- and field-based analyses of hydration status and blood-feeding dynamics

**Figure 5:**
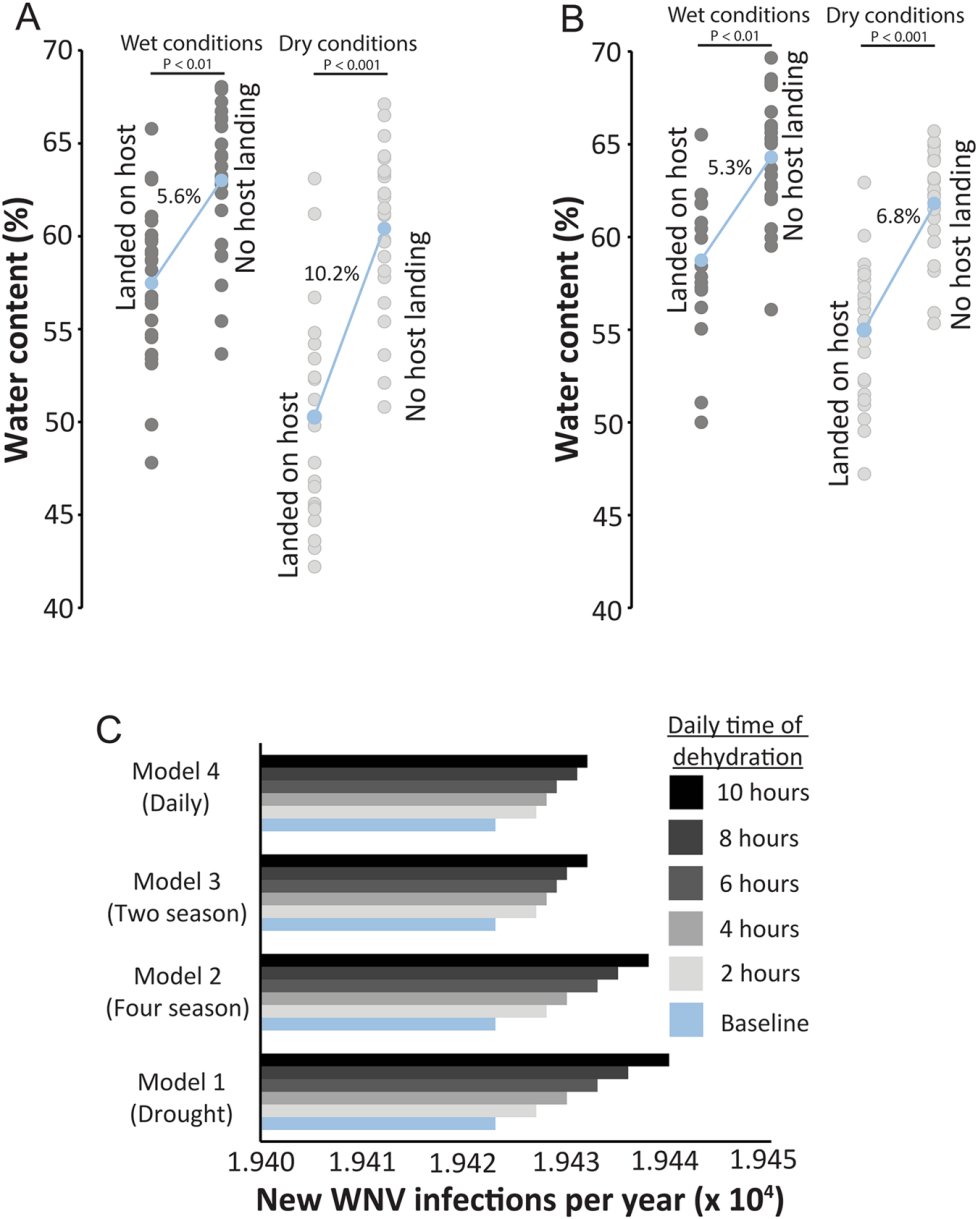
Dry field and laboratory conditions increased host landing and impacted disease transmission based on West Nile virus transmission modeling for *C*. *pipiens* females. (**A**) Body water content (% of total mass) assessment of females attempting to blood feed following 24 hours with and without precipitation from field mesocosm studies. Mean ± SE for 8 replicates at each time point. (**B**) Water content of females attempting to blood feed under laboratory conditions. Mean ± SE of 8 replicates at each time point. (**C**) Predicted transmission of WNV will increase as the result of increased blood feeding due to dehydration exposure. Modeling and wet vs. dry conditions described in Supplemental Materials 1.

To assess if dehydration status impacted blood feeding under field conditions, we designed a mesocosm-based study to assess the water content of the *C*. *pipiens* females attempting to feed on a host mimic. In addition, we examined if mosquitoes held under optimal laboratory conditions would have altered hydration levels in relation to blood feeding. Females were allowed to emerge within an outdoor mesh-enclosed mesocosm or cages within the laboratory and were provided free access to water and sugar. After eight-twelve days, mosquitoes were provided access to an artificial host and mosquitoes that landed and probed over the first 60 minutes were collected. In addition, we collected ~10–20 mosquitoes at the completion of the experiments that did not respond to the host mimic. For the field mesocosm experiments, we performed these studies under two specific weather conditions related to water availability; precipitation had or had not occurred in the last 24 hours. Dry periods yielded a vapor saturation deficit, difference between moisture in the air and amount in air if saturated with water, nearly five- to six-fold higher when compared to periods where rain occurred in the last 24 hours (Fig. S7–S8). Mosquitoes that landed more frequently on a host mimic had reduced water content in comparison to those that did not respond to the host mimic in the laboratory assays and field mesocosms (Fig. 5A). The effect of hydration status on host landing was exacerbated if the preceding 24 hours were dry (Fig. 5A). When examined under similar conditions within the lab, mosquitoes that landed on the host had lower water content compared to those that did not land on a host mimic (Fig. 5B). These results indicate that our laboratory-based studies related to mosquito dehydration exposure are likely to occur under ecological conditions. Importantly, the presence of a sugar and water resource did not fully abate dehydration in mosquitoes, suggesting that even with the presence of fluids a subset of mosquitoes may fail to ingest sugar or water and still be dehydrated.

### Disease modeling based upon increased blood feeding during dehydration

Lastly, to determine if the increased propensity to blood feed yields the potential to prompt higher disease transmission of West Nile virus (WNV), we incorporated the impact of short dehydration bouts into a disease transmission model (Supplemental Materials 1). The northern house mosquito is a common vector for WNV, a flavivirus, throughout North America and Europe^32,33^. We identified that the biting rate on humans is the most sensitive parameter for our baseline model output, cumulated human infections (Fig. S9). Our previously described results indicated that blood feeding rate of *C*. *pipiens* increased following dehydration (Fig. 1), which we subsequently confirmed under different dehydration and rehydration protocols (Fig. S10), which allowed comparable blood-feeding rates for dehydration, control, and rehydrated mosquitoes. Blood-feeding rates were converted to a biting rate based on previous studies^32–34^. With these varying biting rates, we developed four models that incorporate the increased biting rate due to daily dehydration, multiple bouts of dehydration, dehydration in relation to seasonal changes, and the impact of multiple seasons into the potential transmission of WNV (Figs 5C; and S11). Importantly, these models featured dehydration rates based on RH that *C*. *pipiens* are likely to experience in based on conditions recorded near Cincinnati, OH (Figs S7–S8). Under all models, dehydration bouts yielded gradual increases in disease transmission with the largest increase in potential disease transmission after a prolonged period (10 hours) of dehydration.

## Discussion

Mosquitoes must contend with a variety of environmental conditions and dehydration acts as a critical factor in their distribution and survival^20^. This study revealed that bouts of dehydration alter the physiology and behavior of mosquitoes, yielding an increased level of blood feeding, when compared to fully hydrated individuals. This shift in activity and biting rate will likely manifest itself with an increase in the transmission of mosquito-borne diseases following periods of dry conditions. Molecularly, we have provided evidence for the essential role that the breakdown of trehalose into glucose plays in promoting the dehydration-induced phenotype in mosquitoes. Importantly, dehydration-induced phenotypes occur even when mosquitoes have access to sugar and water resources. Specifically, not all females ingest water or nectar often enough to remain fully hydrated and 10% water loss could likely occur within a few hours to induce phenotypic changes. Taken together, these findings indicate that hydration levels can drastically shift mosquito phenotypes and this needs to be accounted for in studies that assess potential disease transmission.

A few studies have assessed metabolomic or transcriptomic changes related to mosquito dehydration^21–23^. In specific, there was an increase in glucose levels following dehydration in *A*. *gambiae* females after dehydration^22^, which was similar to the glucose increase seen in this study. Fluctuations in sugars and polyols have been documented in many insect systems^12,35,36^, which have been suggested to prevent additional body water loss and yield a reduction in potentially damaging interactions among biological molecules. Along with the conversion of trehalose to glucose, we noted a significant, albeit small, decline in glycogen following prolonged dehydration, which yields four to five units of water for each glycogen molecule metabolized^37^. This information indicates that increased glucose levels are a common physiological response following dehydration for mosquitoes and may occur in many other insect systems.

Outside of a potential protective role, there has not been any other role established for this glucose shift during dehydration. Here we show that suppression of metabolic pathways necessary to generate glucose prevented dehydration-induced behaviors. Increased host landing and blood feeding following dehydration are subsequently recovered once the temporal effects of dsRNA targeting *trehalase* have faded. Previous studies have demonstrated that increased carbohydrate oxidation, along with elevated levels of constituents of the TCA cycle, are critical in providing fuel for wing muscles during flight^38–40^. In *A*. *gambiae*, increased expression of genes associated with glycogenolytic processes have been documented when mosquitoes are exposed to dry season conditions^22^, which is comparable to our observed results in *C*. *pipiens*. Of importance, we utilized RNA-seq/metabolomic examination of whole mosquitoes, and more directed studies on specific tissues may yield more precise changes. In specific, our study may have masked even more drastic differences within specific tissues. Nonetheless, whole body RNA-seq combined with the metabolomics indicates a significant shift in carbohydrate metabolism during the course of dehydration. This suggests that the increased sugar and amino acids may act beyond their roles in protection from stress and liberation of free water to potentially serve as an energy source. Such utilization of these nutrient stores would support behavioral shifts that could increase blood feeding as a source of water.

The rate of dehydration is influenced by both the temperature and relative humidity, where low RH and high temperature yield the highest water loss rates. Under conditions that induce dehydration rapidly (33% RH, 35 °C), water loss reaches a critical threshold of 10% within ~4 hours and under less severe conditions (75% RH, 25 °C) this threshold takes ~12–24 hours to reach. Based on weather patterns that we observed in this study, conditions necessary to increase blood feeding could potentially happen daily when precipitation is low. Importantly, the most drastic effects on glucose concentration, activity, and blood feeding occurred at a slower rate of dehydration and can occur after only a 10% water loss. This suggests that the findings from this study are ecologically relevant, and critical in the analysis of the dehydration-induced phenotypes in mosquitoes. Even if mosquitoes retreat to more favorable microhabitats^9^, it is likely that dehydration will still play a role in blood feeding if no liquid water is available to drink. This retreat is likely mimicked by activity changes observed when mosquitoes were held at 75% RH when water was present, but water ingestion was not possible. These favorable microhabitats will only partially suppress, and likely extend, dehydration-induced phenotypes unless liquid water or nectar is available for ingestion.

Of particular interest is that we show that the water content of mosquitoes landing on a host mimic is lower than those not landing based on both mesocosm and laboratory studies. This occurred despite mosquitoes having access to both sugar and water sources, which should allow individuals to remain hydrated and abate dehydration-induced phenotypes. This is likely due to hydration levels of mosquitoes that are not uniform (difference of 10–15%), even under stable laboratory conditions with free access to liquids. Indeed, the water content of female *C*. *pipiens*, although when averaged was ~65%, varied from 56–67% within our colonies (this study,^19^). Thus, individual mosquitoes, even with access to water resources, may have 10–15% lower water content than other conspecifics. The importance of demonstrating that hydration levels can alter host landing under stable, rather than drought-like, laboratory conditions is that dehydration-induced phenotypes may not require a distinct dry period. Instead, drought periods will only exacerbate shifts in phenotypes associated with dehydration, such as increased activity and blood feeding.

Along with the utilization of a bloodmeal or water for rehydration, nectar represents a major source of water that could be utilized by mosquitoes to remain hydrated^41,42^. Even though nectar consumption during periods of drought would likely occur, its availability also declines during periods of low water availability^43–45^. These conditions are likely to be exacerbated by climate change^46^. This suggests that nectar, similar to free water sources, may not be as readily available under drought conditions^43–45^. Furthermore, even without extended drought periods, mosquitoes likely fail to utilize sugar and water resources over short periods, which will yield dehydration-induced phenotypes of increased blood feeding. This failure to ingest fluid is likely the case as our field mesocosm and laboratory studies showed that the subset of mosquitoes that host landed were more dehydrated than those remaining off host, despite free access to sugar and water.

The increased host landing and blood feeding following dehydration is one of the most intriguing results from this study. Increased levels of general activity are likely a major contributing factor to the higher propensity to interact with a host, but there could be other underlying mechanisms. In specific, we demonstrated that dehydrated mosquitoes are more likely to land on a warm host mimic. Location of a host is accomplished through mechanisms that integrate multiple sensory cues to locate, land, and probe a host^47–51^. Our host mimic does not generate increased CO_2_, rather only increased heat in conjunction with basic components of perspiration and increased local relative humidity. This suggests that there are multiple potential sensory mechanisms that could be tuned in mosquitoes by dehydration, including increased temperature detection^52^, humidity detection^17,47,53^, host volatile detection^47–51^, or a combination of thermal-humidity-host cue dynamics. Thus, detection thresholds for sensory cues will need to be examined more in-depth following bouts of dehydration in mosquitoes.

Our modeling studies suggest that periods of dehydration are likely to impact the transmission of WNV based on increased blood-feeding rates. Although our increased transmission rates are minimal (~20–30 news cases per year within a specific area), this would present nearly three- to four-fold more cases in most states within the United States based upon cases reported to the CDC^54^. Of importance, our analyses only took into account altered blood feeding, excluding other factors such as impact on survival, longevity, and reproduction that could shift as a result of dehydration bouts^1,42,55,56^. Viral development and general vector competence of mosquitoes might be altered by the hydration status, similar to temperature^57–59^. Future studies will be necessary to fully elucidate the impact of mosquito dehydration on viral transmission based upon the combination of increased mosquito blood feeding during dehydration along with other altered biological parameters.

In conclusion, bouts of dehydration alter carbohydrate metabolism, which likely supports shifts in mosquito activity and prompts increased blood feeding. This blood feeding is not the direct result of starvation, but likely serves as a mechanism for immediate rehydration. Under dry periods it is likely that rehydration through host feeding could be more feasible than ingestion of freestanding water or nectar, since temporal water pools may be difficult to locate in some environments and nectar production is reduced. This increased propensity of blood feeding will likely increase the potential for disease transmission, due to a higher biting rate under dry periods with low precipitation. Recent studies have indicated that the transmission rates of West Nile virus are impacted by dry periods, where drought has been identified as one of the most critical factors underlying WNV outbreaks^60–65^. Specifically, there have been four mechanisms where dehydrating conditions have been suggested to impact disease transmission dynamics. First, periods of drought likely bring mosquitoes and wild animals in close contact due to the scarcity of water sources^66^. Second, dry conditions will concentrate nutrients in water pools^61,62^, providing the resources necessary for larval development. Third, periodic drying will suppress the levels of predators and other competitors and allow mosquitoes to proliferate^67^. Fourth, lack of rain will allow for water to pool in storm drains^68^, which yield temporal water pools for mosquito development. In this study, we establish a fifth major dehydration-induced factor that will impact disease transmission, where bouts of dehydration impact the physiology and behavior of mosquitoes prompting increased blood feeding. This shifted biting rate, due to dehydration, needs to be accounted for in studies that seek to predict the prevalence and potential outbreaks of mosquito-borne diseases such as West Nile, Zika, or Dengue viruses.

## Materials and Methods

### Mosquito husbandry

Mosquitoes were maintained in a climate-controlled facility at the University of Cincinnati. Ambient temperature was held between 24–28 °C and relative humidity (RH) was held between 70–80%. A 12–12 h light-dark photoperiod was maintained for the duration of the experiment. For each species, eggs were harvested after oviposition. Post-emergence, larvae were separated into tanks of 75–85 individuals per 0.5 L water to control for density during maturation. Larvae were fed finely ground fish food (Tetramin, Goldfish Flakes) with added yeast extract (Difco, BD 210929). Adults were kept in 12″ x 12″ x 12″ mesh cages with unrestricted access to clean water and a 10% sucrose solution. Females were used in all subsequent experiments and were 10–14 days post emergence. Three species were used in this study: *Aedes aegypti* Benzon, *Culex pipiens* Buckeye^69^, and *Anopheles quadrimaculatus* Benzon. The colony of *C*. *pipiens* was establish in 2000 from mosquitoes collected in Columbus, OH, supplemented with new field collections in 2008 and 2012 to maintain the ability to undergo diapause, and maintained at the University of Cincinnati since 2014. The *A*. *aegypti* and *A*. *quadrimaculatus* were acquired from Benzon Research (Carlisle, PA, USA). The strains of *A*. *aegypti* and *A*. *quadrimaculatus* originated from a colony in Gainesville, FL, USA in 1993 and 2001, respectively.

### Dehydration experiments

Mosquitoes were dehydrated by placing groups of 20 mosquitoes in a 50 ml centrifuge tube covered with mesh. These tubes were placed at 0% RH or 75% RH and removed after either 10–15% water loss (17–19 hours at 75% RH or 4–6 hours at 0% RH) or 20–25% water loss (35–37 hours at 75% RH or 9–11 hours at 0% RH). Control mosquitoes were either held at 100% RH (generated with deionized water) or exposed to dehydrating conditions (75% RH, generated with saturated NaCl solutions) and allowed access to free water to remain fully hydrated. In addition, a subset of dehydrated mosquitoes were moved to 100% RH with access to free water to determine if the phenotypes induced by dehydration could be reversed when water content was recovered. A schematic of the dehydration experiment is included as Figure S12. This allowed comparisons among dehydrated, fully hydrated, and fully hydrated under dehydrating conditions to elucidate the effect of body water content reduction versus exposure to dehydrating conditions, which has been lacking in other studies on the response of arthropods to dehydration.

### RNA-seq and metabolomic analyses

RNA-seq analyses were conducted on dehydrated (20% loss) and fully hydrated mosquitoes. RNA was extracted with Trizol, treated to remove DNA, and cleaned based upon manufacturers’ methods (Invitrogen, 15596018; Qiagen, 74104). Two independent biological libraries were prepared and sequencing was conducted at the Cincinnati Children’s Hospital Medical Center DNA Sequencing and Genotyping Core. Reads were mapped to predicted genes from the *Culex quinquefasciatus* genome (Gene set version 2.1) set using CLC Genomics (CLC Bio) based upon methods used in Benoit *et al*.^70^ and Rosendale *et al*.^36^. Differentially expressed genes were determined using transcripts per million (TPM) with P values corrected using the Bonferroni method to reduce false discovery. Gene Ontology (GO) terms were identified with Blast2GO^71^ and g:Profiler^72^. Specific KEGG pathways were examined for enrichment through the use of Gene Set Enrichement Analyses (GSEA)^73,74^. Quantitative PCR (qPCR) was utilized to validate differences in transcript abundance and conducted according to previous studies^36^ and described in the supplemental materials (Supplemental materials 1; Table S3). RNA-seq data sets have been deposited to the NCBI Sequence Read Archive (Bioproject PRJNA418113).

Metabolomics were conducted on mosquitoes after 18–20 hours of dehydration at 75% RH (Dehydrated) or 100% RH (Control) according to methods used by Rosendale *et al*.^36^. Briefly, mosquitoes were collected and dried at 0% RH and 50 °C for five to seven days (no daily changes in mass noted) to establish the dry mass, which was used to standardize each sample. Eight replicates of 30 to 40 mosquitoes were collected and analyzed for the control and dehydrated samples.

### Gene knockdown

*trehalase* levels were suppressed through the use of dsRNA injection (1.0–1.5 µg/µl), which was generated and injected according to methods developed previously^10,75^. CO_2_-anesthetized females were treated at 3 days post-emergence by injection into the thorax with a pulled glass capillary needle. Knockdown was validated through the use of qPCR^36^ and a trehalase activity assay^76,77^. Specific details of the modeling are included in the supplemental information (Supplemental Materials 1).

### Survival analyses

Mosquito survival assays were conducted to determine the time until death. Three conditions were examined; dehydration, dehydration-inducing conditions with access to free water, and exposure to high RH (100% RH) under similar conditions as listed above for the dehydration experiments. Mosquito death was determined when mosquitoes failed to respond to stimulus and move. Differences in survival were assessed based on the LD_50_ determined with a Kaplan-Meier survival analyses (JMP, SAS software). Three groups of 50 mosquitoes were used for each treatment.

### Nutrient reserves

Levels of mosquito nutrient reserves were measured using spectophotometric methods on a Bioteck microplate reader (Synergy HT) according to previous studies^1,31,78–80^. Briefly, mosquitoes were killed by moving treatment tubes to −70 °C. Three mosquitoes were combined and homogenized (6.50 m/s for 10 cycles with 5 sec break between cycles) with the use of a bead blaster in 250 µl of 2.0% Na_2_SO_4_. Soluble sugars were extracted using a 1:1 chloroform/methanol mixture. Glycogen was quantified with an anthrone (Sigma-Aldrich, 319899) assay^79^. Trehalose (Sigma-Aldrich, T9531) and glucose (Sigma-Aldrichm GAHK20) assays were conducted as described in Liu *et al*.^75^ Dry masses were determined for each group with no significant differences noted at 0, 18, or 36 h.

### Landing and blood-feeding assays

The number of mosquitoes that landed on the host was determined based on techniques modified from Barnard *et al*.^81^. Mosquitoes (20–30) were released into a 60 × 60 × 60 cm cage (Bugdorm, BD6S610) and allowed two hours to equilibrate. An artificial host (Hemotek) was covered three times with Parafilm, filled with water, coated with 100 µl artificial eccrine perspiration (Pickering, 1700–0022), heated to 37 °C, and attached to the top of the cage. Mosquitoes that landed and remained on the artificial host for at least 5 seconds were used to differentiate incidental contact from exploratory (foraging) contact. The number of mosquitoes making foraging contact was counted at five-minute intervals for 60 minutes. A subset of landing assays were conducted in a blinded fashion, where the individual counting landing events did not know the hydration status of the mosquitoes to ensure a lack of bias. Blood feeding was measured using the same apparatus and setup except the Parafilm was pulled thin to allow successful probing and the feeding disk was filled with citrated chicken blood (Pel-Freez, 33130-1). Relative water loss was determined for a subset of mosquitoes (N = 10) prior to blood-feeding assay to determine level of dehydration.

### Activity assays

General activity of mosquitoes was assessed using a Locomotor Activity Monitor 25 (LAM25) system (TriKinetics, Waltham, MA) based on methods previously developed for mosquitoes^82^. Briefly, females 7–10 days post-emergence (mated, but not blood fed) were moved into 25 × 150 mm clear glass tubes. Flight activity was recorded as the number of times a mosquito passed across an infrared beam. All recordings were conducted under continual light cycles to prevent confounding effects of light:dark transition on the dehydration response, which has been noted in other studies^82,83^. Four groups were analyzed: mosquitoes held under 75% RH (dehydrated), those held at 100% RH (fully hydrated), mosquitoes at 75% RH with access to free water (fully hydrated, but exposed to dehydration-inducing conditions), and lastly mosquitoes at 75% RH featuring the apparatus for free water that is blocked to free drinking (dehydrated with presence of free water, but ingestion is not possible).

### Lab colony and field-based mesocosm studies on hydration status of blood-feeding mosquitoes

To determine if baseline differences in hydration can drive mosquito feeding, we examined if there is a difference in the hydration status of individuals that blood feed under laboratory or semi-field conditions (mesocosm studies). For field-based studies, lab-reared mosquitoes were released into a 6′ x 6′ x 6′ mesh-covered cage (Bioquip, 1406 C) at the University of Cincinnati Center for Field Studies (39.2849 N, 84.741 W). Water and 10% sucrose solution were provided *ad libitum* and changed every two days with the last change occurring 24 hours before the testing. A host mimic as previously described was introduced in the evening and mosquitoes that landed on the host mimic were collected and immediately frozen. Conditions where precipitation occured in the preceding 24 hours were required for a wet period. In addition, eight to ten mosquitoes that did not land of the host mimic were collected. Water content of these mosquitoes was determined according to Benoit and Denlinger^19^. For laboratory studies, the host mimic was added to a small lab colony (~50 mosquitoes) and mosquitoes that did or did not land on the host mimic were collected. Specific details on mesocosm design and weather conditions along with the laboratory colony assays are included in the supplemental information (Supplemental Materials 1).

### Modeling of disease transmission

Modeling of WNV transmission was developed based on previous studies^32–34^. We applied sensitivity analysis on our model and identified important parameters that dominated our model output. We modified the biting rates under each model based upon proportional changes in blood-feeding that examined the impact of dehydration, dehydration with access to water, and those experiencing no dehydration stress on blood feeding (Fig. S9). Specific details of the modeling are included in the supplemental information (Supplemental Materials 1).

### Statistics and replication

Replicates throughout the experiments are biologically independent and distinct samples. Sample sizes are listed in the methods section or the appropriate figure legend. Statistical significance between treatments and controls is indicated within each figure and/or in the figure legend. Statistical tests are listed within the respective figure legends. All statistical analyses were performed using JMP version 11 (SAS) or R-based packages (R version 3.1.2).

### Availability of data and materials

All data generated or analyzed during this study are included in this published article, within the supplement, or have been deposited to the NCBI Sequence Read Archive.

## Electronic supplementary material

Table S1

Table S2

Table S3

Supplementary Figure
