# Supplementary material for: Dehydration prompts increased activity and blood feeding by mosquitoes
Source: Sci Rep. 2018 May 1;8:6804. doi: 10.1038/s41598-018-24893-z (PMC5931509; doi:10.1038/s41598-018-24893-z)
Supplement: Supplementary file 4 — Supplementary Figure [file 41598_2018_24893_MOESM4_ESM.pdf]

Supplementary information for:

Dehydration prompts increased activity and blood feeding by mosquitoes

Richard W. Hagan[a,1], Elise M. Didion [a,1], Andrew E. Rosselot [a,1], Christopher J. Holmes [a,1], Samantha C. Siler[a], Andrew J. Rosendale[a], Jacob M. Hendershot[a], Kiaira S. B. Elliot[a], Emily C. Jennings[a], Gabriela A. Nine[a], Paula L. Perez[a], Alexandre E. Rizlallah[a], Miki Watanabe[c], Lindsey E. Romick-Rosendale[c], Yanyu Xiao[b], Jason L. Rasgon[d], and Joshua B. Benoit[a,2]

[a]Department of Biological Sciences, University of Cincinnati, Cincinnati, OH 45221 USA.

[b]Department of Mathematical Sciences, University of Cincinnati, Cincinnati, OH 45221 USA.

[c]Division of Pathology, Cincinnati Children's Hospital Medical Center, Cincinnati, OH 45229 USA.

[d]Department of Entomology, Pennsylvania State University, Center for Infectious Disease Dynamics and Huck Institutes for Life Sciences, Pennsylvania State University, University Park, PA 16802 USA.

[1]Authors contributed equally

[2]Author for correspondence

Joshua B Benoit

Department of Biological Sciences,

University of Cincinnati, Cincinnati, OH

Email: joshua.benoit@uc.edu

Phone: 513-556-9714

Quantitative PCR (qPCR)

qPCR was utilized as validation of the RNA-seq studies and in measuring the expression of trehalase following dsRNA injection. RNA was extracted with Trizol, treated to remove DNA, and cleaned based upon manufacturers' methods. Complementary DNA (cDNA) was generated from the RNA with the use of a DyNAmo cDNA Synthesis Kit (Thermo Scientific, F-470L). Each cDNA synthesis reaction consisted of 400 ng RNA, 50 ng oligo(dT) primers and reaction buffer (dNTPs + M-MuLV RNase H+ reverse transcriptase). qPCR reactions contained KiCqStart SYBR Green qPCR ReadyMix (Sigma Aldrich, KCQS07), 300 nmol l^{-1} forward and reverse primers, cDNA diluted 1:20, and nuclease-free water. Primers were designed with Primer3 (Table S3). qPCR reactions were conducted using an Eco Realtime PCR System (Illumina). Reactions consisted of polymerase activation for 3 min at 95°C followed by 50 cycles of denaturation for 10 s at 95°C, annealing/extension for 30 s at 55°C and denaturation for 10 s at 95°C. After amplification, a melt curve analysis was performed from 55 to 95°C with 0.5°C increments every 15 s. Each sample was analyzed in triplicate and average quantificationcycle (C_q) was established. Expression levels for the gene of interest were normalized against *alpha-tubulin*, which did not vary expression during dehydration, using the ΔΔCT. The fold change during dehydration was determined relative to the control (fully hydrated) mosquito or in respect to the control knockdown.

Mesocosm design and weather conditions.

Mesocosm-based studies were conducted at the University of Cincinnati Center for Field Studies (39.2849N, 84.741W). Three 6' x 6' x 6' mesh-covered cages (Bioquip, 1406C) were placed within 50 m of the weather station (HOBO, Onset, RX3000). Two small structures (Aspen PetBarn Dog houses, 125282) with a cinderblock added as a resting site for the mosquitoes were placed within the tent as resting sites for the mosquitoes. Tents were constructed early May 2016 and were not used for testing until June to allow for equilibration with the environment. Water was provided freely within two plastic containers (Pioneer Plastics, 149C) that were refilled as needed. A sugar source (10% sucrose) was provided and changed two-three times per week. The sugar solution was stored within a Vaseline-lined glass beaker to prevent ant infestation. Lab reared pupae were placed into the tents and allowed to emerge. Testing of mosquitoes was conducted when the mosquitoes were 10-14 days of age and weather conditions met specific criteria. Mosquitoes were examined under wet conditions if precipitation occurred 24 hours prior to mosquito collection (Figure S7-S8). Four specific collection times met these criteria One collection time was not utilized as a severe thunderstorm damaged the tent and impacted the placement of the artificial host. Dry collection periods were when no rain occurred 24 hours prior to testing. Five specific collection times met these criteria. Two were not used (spider establishment within a tent eliminated mosquitoes and no mosquitoes were collected). Mosquitoes were allowed access to a host mimic (Hemotek) as described in the main paper and those that landed were collected and water content was determined as in Benoit and Denlinger [1].

Individuals were collected from within the tent that did not land on a host and water content was determined as before.

Laboratory blood feeding and hydration experiments

Mosquitoes were maintained as described in the main methods. After 10-14 days following emergence, the landing assay apparatus (described in main text) was added and mosquitoes that landed were collected. Water content was determined as in Benoit and Denlinger [1]. Individuals were collected from within the tent that did not land on a host and water content was determined.

Trehalase activity

Trehalase activity was examined as previously described [2] with modifications. Five female mosquitoes were homogenized at speed of 6.50 m/s for 10 cycles with 5 sec break between in phosphate buffered saline (PBS, pH 7.2) using a Beadblaster 24 (Benchmark Scientific). The homogenate was centrifuged at 1000g for 15 min at 4 °C to remove cuticle debris. The supernatant (60 µl) was incubated with 165 µl of PBS and 75 µl of 40 mM trehalose (Sigma-Aldrich, T9531). After 1 h, each sample was centrifuged at 12,000 g for 10 minutes at 4°C. Total trehalase activity was determined based on the amount of trehalose that was converted to glucose using a glucose (GO) assay kit (Sigma-Aldrich, GAGO20) using 10 µl of supernatant. Trehalase activity was examined between dehydrated and following knockdown of trehalase using dsRNA sample. Each treatment was replicated four times with three technical replicates.

Modeling for West Nile Virus transmission

We developed a basic mathematical model for West Nile Virus infection and pathogen transmission among humans (H), birds (B) and female mosquitoes (M) based on previous studies [3-5]. Three compartments for susceptible (S), exposed (E) and infectious (I) groups were considered for humans, birds and female mosquitoes, respectively. We used the subscript to represent the species, i.e. S_H is the total susceptible human population. In the absence of disease, the total populations of birds and female mosquitoes followed the standard logistic growth. Our basic model system is expressed as follows,

$$
\begin{aligned}
\frac{\mathrm{d}S_H(t)}{\mathrm{d}t} &= \eta_H - a_{HM}\beta_{HM}(t)S_H(t)I_M(t) - d_H S_H(t) + \gamma_H I_H(t),\\
\frac{\mathrm{d}E_H(t)}{\mathrm{d}t} &= a_{HM}\beta_{HM}(t)S_H(t)I_M(t) - (d_H + d_{HH} + \varepsilon_H)E_H(t),\\
\frac{\mathrm{d}I_H(t)}{\mathrm{d}t} &= \varepsilon_H E_H(t) - (d_H + d_{HH} + \gamma_H)I_H(t),\\
\frac{\mathrm{d}S_M(t)}{\mathrm{d}t} &= b_M(S_M(t) + E_M(t) + I_M(t)) - \frac{(b_M - d_M)(S_M(t) + E_M(t) + I_M(t))^2}{K_M(t)}\\
&\quad - a_{MB}\beta_{BM}(t)S_M(t)I_B(t) - d_M S_M(t),\\
\frac{\mathrm{d}E_M(t)}{\mathrm{d}t} &= a_M\beta_{BM}(t)S_{MB}(t)I_B(t) - (d_M + \varepsilon_M)E_M(t), \qquad (1)\\
\frac{\mathrm{d}I_M(t)}{\mathrm{d}t} &= \varepsilon_M E_M(t) - d_M I_M(t),\\
\frac{\mathrm{d}S_B(t)}{\mathrm{d}t} &= \eta_B + b_B(S_B(t) + E_B(t) + I_B(t)) - \frac{(b_B - d_B)(S_B(t) + E_B(t) + I_B(t))^2}{K_B(t)}\\
&\quad - a_{BM}\beta_{BM}(t)S_B(t)I_B(t) - d_B S_B(t) + \gamma_B I_B(t),\\
\frac{\mathrm{d}E_B(t)}{\mathrm{d}t} &= a_{BM}\beta_{BM}(t)S_B(t)I_M(t) - (d_B + d_{BB} + \varepsilon_B)E_B(t),\\
\frac{\mathrm{d}I_B(t)}{\mathrm{d}t} &= \varepsilon_B E_B(t) - (d_B + d_{BB} + \gamma_B)I_B).
\end{aligned}
$$

In Table 1, we provided detailed explanations of parameters for system (1).

Table 1. Model parameters (daily). H: humans; B: birds; M: mosquitoes.

Notation	Name	Selected values
a_{HM}	Effective transmission rate (M->H)	0.1
a_{MB}	Effective transmission rate (B->M)	0.16 [2]
a_{BM}	Effective transmission rate (M->B)	0.88 [2]
$\beta_{HM}(t)$	Biting rate (M->H)	Variable (refer to Table 2) Baseline: 0.09 [3]
$\beta_{BM}(t)$	Biting rate (M->B)	Variable (refer to Table 2) Baseline: 0.09 [2]
η_H	Constant recruitment rate (H)	0.3
η_B	Migration rate (B)	12
d_H	Natural death rate (H)	1/65/365
d_B	Natural death rate (B)	0.011 [4]
d_M	Death rate (M)	0.001 [2]
d_{HH}	WNVs induced death (H)	0.00014 [4,5]
d_{BB}	WNVs induced death (B)	0.143 [2]
b_M	Birth rate (M)	0.02
b_B	Birth rate (B)	0.01
$K_M(t)$	Carry capacity (M)	Baseline 1800, seasonal
$K_B(t)$	Carry capacity (B)	Baseline 1200, seasonal
k_M	Seasonal elasticity (M)	0.04
b_B	Seasonal elasticity (B)	0.04
γ_H	Recovery rate (H)	1/14 [3]
γ_B	Recovery rate (B)	0.001
ε_H	Probability of becoming infectious (H)	1/14 [3]
ε_M	Probability of becoming infectious (M)	0.1 [2]
ε_B	Probability of becoming infectious (B)	0.1
T_1	Dehydration daily period	24 hours
T_2	Seasonal period	365*24 hours

We performed sensitivity analysis on system (1) using the output, cumulated number of human infections. We applied Latin hypercube sampling and partial rank correlation coefficient analysis on some important parameters, such as biting rates and birth and death rates of female mosquitoes, which are likely to vary under dehydration stress. We found that the cumulated number of human infections is highly sensitive to the biting rate and effective transmission rate from mosquito to humans and less sensitive to the birth and death of mosquitoes, shown in Figure 1. Therefore, when we quantitatively evaluate the impact of dehydration stress on the cumulated number of human infections, we can focus on the varied biting patterns and that slight changes in birth and death rates of mosquito brought by dehydration will likely have much less impact

To examine the effect of multiple bouts of dehydration and seasonal changes on disease dynamics of WNVs transmission, we examined five models based on system (1) with different functions for biting rates, β_{HM} and β_{BM}, and/or carrying capacities for female mosquitoes and birds, $K_M(t)$ and $\mathrm{K}_B(t)$. Biting rates changes daily due to bouts of dehydration (Figure S11) varying from 2 hours to 10 hours starting from 14:00 each day. The availability of sugar and water was used to simulate warm seasons, while the availability of rehydration treatment was adopted to simulate environment with relative humdities near saturation. Biting rates also varies during different seasons. Additionally, we considered that no biting activity would occur in winter as *C. pipiens* is in dormancy. Notice that the biting rates we obtained from the

experiments are higher than the corresponding values in the references [3,4], here we considered the biting rate, in the case without dehydration, sugar and water, and rehydration treatment, to be 0.09 [3,4] as the baseline level and then normalized other experimental difference in biting rates for our models, shown in Table 2. In our baseline model, no dehydration and seasonal effect were modeled. In model 1, we only examined the effect of multiple bouts of dehydration. We gradually emphasized the seasonal effect in models 2 to 4. Comparisons between five models are presented in Table 3 and shown in Figure S12. The annual cumulative infected population calculated by each model for different duration of daily dehydration is calculated in Table 4.

Table 2. Mosquito biting rates obtained in various experiments. (Unit: bite per day)

Biting rate	Experimental Value	S-Dev	Converted Value	Dehydration	Sugar and Water	Rehydration
β_{11}	0.22	0.12	0.09[1]	No	No	No
β_{12}	0.24	0.14	0.098	No	Yes	No
β_{21}	0.86	0.21	0.352	Yes	No	No
β_{22}	0.32	0.24	0.131	Yes	No	Yes
β_{31}	0.35	0.14	0.143	Yes	Yes	No
β_{32}	0.26	0.23	0.106	Yes	Yes	Yes

Table 3. Model settings and comparisons.

Model	Biting rates ($\beta_{HM}(t)$ and $\beta_{BM}(t)$, denote by '$\beta_{*}(t)$')	Capacity
Baseline model	No dehydration stress, constant β_{11}	Constant

Model 1 (Daily)	Daily dehydration routine, periodic function with a period of 24 hours. $\beta_*(t)=\begin{cases}\beta_{21}, & \text{t} \in \text{Dehydration period (DP)} \cap \{\text{spring, summer, fall}\} \\ \beta_{11}, & \text{t} \in \text{Non dehydration period } (NDP) \cap \{\text{spring, summer, fall}\}, \\ 0, & \text{t} \in \{\text{winter}\}.\end{cases}$	Constant
Model 2 (Two Seasons)	Daily dehydration routine and seasonal impact on temperature. $\beta_*(t)=\begin{cases}\beta_{31}, & \text{t} \in \text{DP} \cap \{\text{late spring, summer, early fall}\}, \\ \beta_{12}, & \text{t} \in NDP \cap \{\text{late spring, summer, early fall}\}, \\ \beta_{21}, & \text{t} \in \text{DP} \cap \{\text{early spring, late fall}\}, \\ \beta_{11}, & \text{t} \in N\text{DP} \cap \{\text{early spring, late fall}\}, \\ 0, & \text{t} \in \{\text{winter}\}.\end{cases}$	Seasonal
Model 3 (Four seasons)	Daily dehydration routine and seasonal impact on temperature. $\beta_*(t)=\begin{cases}\beta_{31}, & \text{t} \in \text{DP} \cap \{\text{summer}\}, \\ \beta_{12}, & \text{t} \in NDP \cap \{\text{summer}\}, \\ \beta_{32}, & \text{t} \in \text{DP} \cap \{\text{fall}\}, \\ \beta_{12}, & \text{t} \in N\text{DP} \cap \{\text{fall}\}, \\ \beta_{22}, & \text{t} \in \text{DP} \cap \{\text{spring}\}, \\ \beta_{11}, & \text{t} \in N\text{DP} \cap \{\text{spring}\}, \\ 0, & \text{t} \in \{\text{winter}\}.\end{cases}$	Seasonal
Model 4 (Drought)	Daily dehydration routine and seasonal impact on temperature and humidity. $\beta_*(t)=\begin{cases}\beta_{31}, & \text{t} \in \text{DP} \cap \{\text{summer}\}, \\ \beta_{12}, & \text{t} \in NDP \cap \{\text{summer}\}, \\ \beta_{32}, & \text{t} \in \text{DP} \cap \{\text{late spring, early fall}\}, \\ \beta_{12}, & \text{t} \in N\text{DP} \cap \{\text{late spring, early fall}\}, \\ \beta_{22}, & \text{t} \in \text{DP} \cap \{\text{early spring, late fall}\}, \\ \beta_{11}, & \text{t} \in N\text{DP} \cap \{\text{early spring, late fall}\}, \\ 0, & \text{t} \in \{\text{winter}\}.\end{cases}$	Seasonal

Table 4. Annual cumulated infections simulated by each models.

Cumulated infections (10^4)		Daily dehydration period				
		2 hours	4 hours	6 hours	8 hours	10 hours
Model	Model 1	1.9470	1.9473	1.9477	1.9480	1.9484
	Model 2	1.9470	1.9472	1.9474	1.9476	1.9479
	Model 3	1.9469	1.9470	1.9471	1.9472	1.9473
	Model 4	1.9469	1.7527	1.9471	1.9472	1.9474
	Baseline Model*	1.9465				

* No daily dehydration period

Reference:

[1] Benoit., J. B. & Denlinger, D. L. Suppression of water loss during adult diapause in the northern house mosquito, *Culex pipiens*. *J. Exp. Biol.* **210**, 217-226. (2007).

[2] Tang, B., Yang, M., Shen, Q., Xu, Y., Wang, H., & Wang. S. Suppressing the activity of trehalase with validamycin disrupts the trehalose and chitin biosynthesis pathways in the rice brown planthopper, *Nilaparvata lugens*. *Pest. Biochem. Physiol.* **137**, 81-90 (2017).

[3] Wonham, M. J., de-Camino-Beck, T., & Lewis, M. A., An epidemiological model for West Nile virus: invasion analysis and control applications. *Proc. Royal Soc. B.* **271**, 501–507 (2004).

[4] Bowman, C., Gumel, A. B., van, d. D., Wu, J. & Zhu, H., A mathematical model for assessing control strategies against west nile virus. *Bull. Math. Biol.* **67**, 1107-33, (2005).

[5] Chen, C., Huang, J., Beier, J. C., Cantrell, R. S., Cosner, C., Fuller, D. O., Zhang, G. & Ruan, S., Modeling and control of local outbreaks of West Nile virus in the United States. *Discr. Cont. Dynam. Syst. – Ser. B*. **21**, 2423-2449, (2016).

[6] Centers for Disease Control and Prevention (CDC),West Nile Virus, March 26, 2014. Available from:http://www.cdc.gov/westnile/index.html.

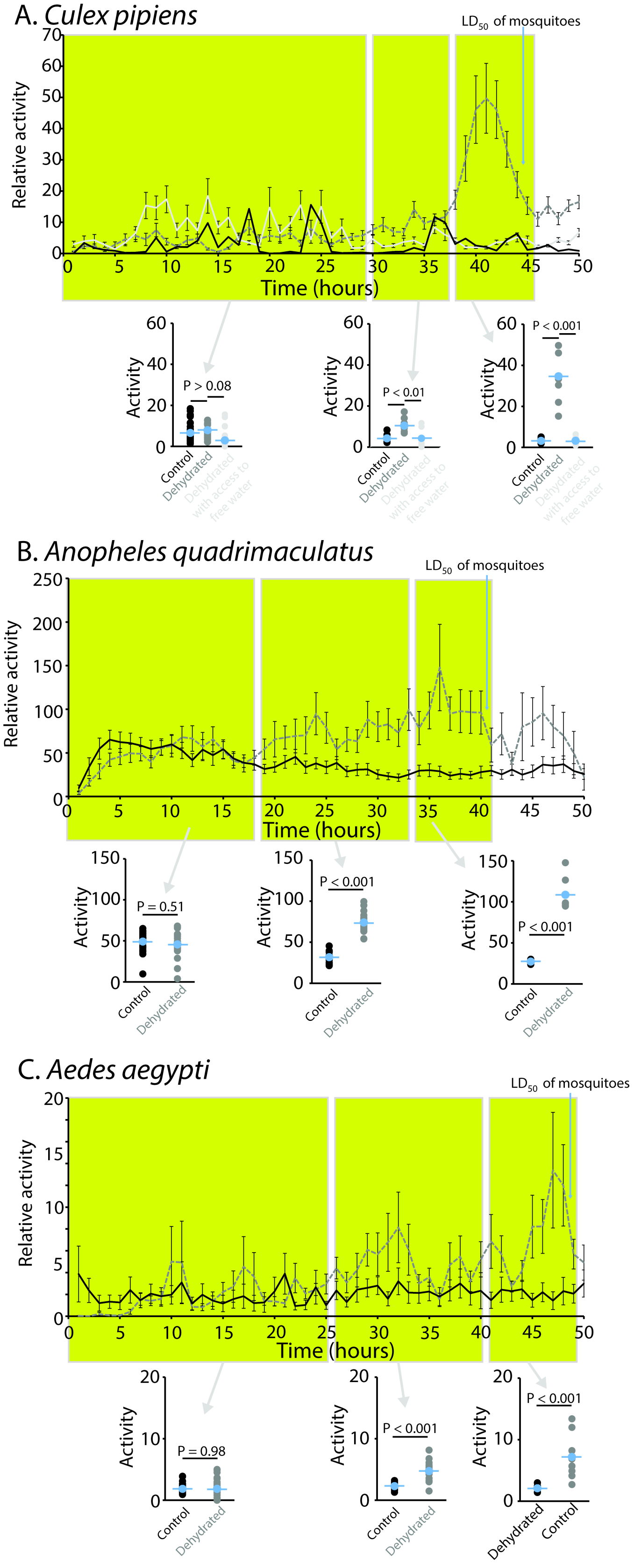

Figure S1: Activity through the course of dehydration for *Culex pipiens*, *Aedes aegypti*, and *Anopheles quadrimaculatus*. Each time course represents the mean ± SE represents 48 mosquitoes. Statistical analyses were conducted by a t-test. Specific shaded areas are highlighted by individual plots to allow comparison between different periods of dehydration.

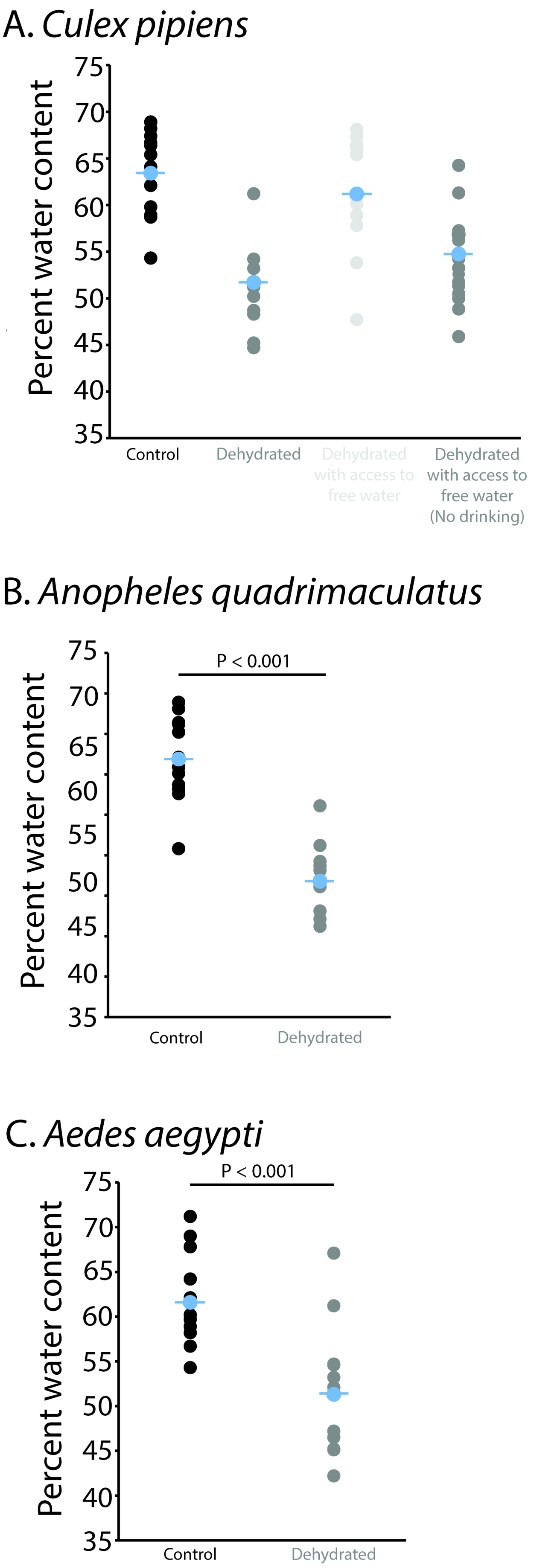

<u>Figure S2</u>: Dehydration status of mosquitoes after 40 hours at 75% RH. Statistical analyses were conducted by a one-way ANOVA or t-test followed by Tukey's post-hoc analysis.

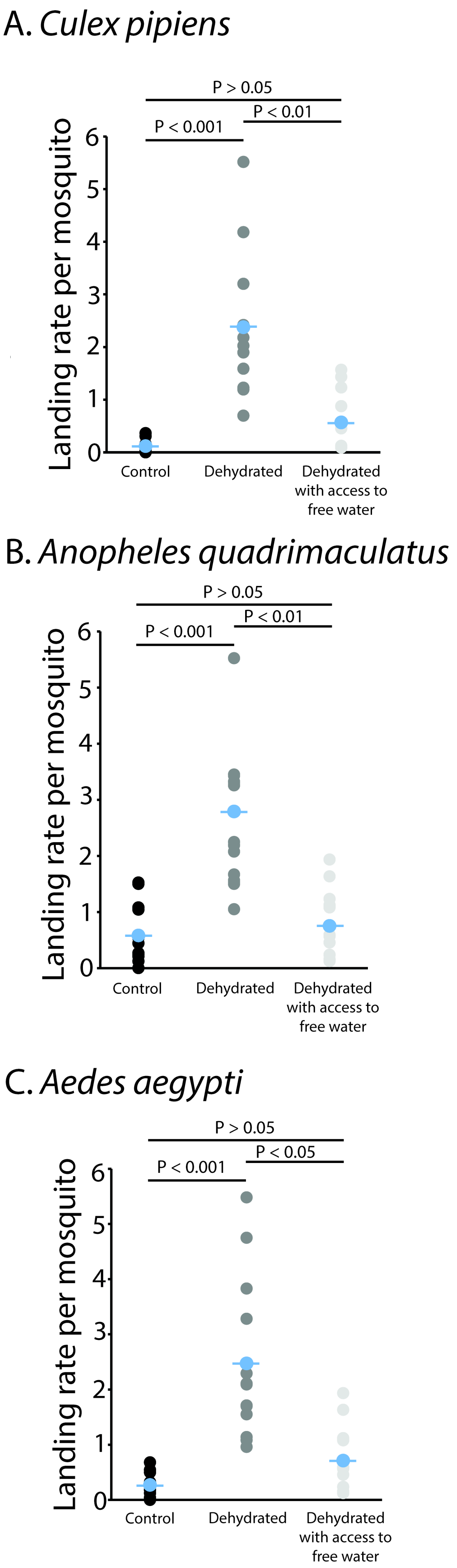

Figure S3: Number of landing events per mosquito over the course of one hour. Mean ± SE represents 11-13 independent replicates of 30-40 mosquitoes. Statistical analyses were conducted by a one- or two-way ANOVA followed by Tukey's HSD post-hoc analysis.

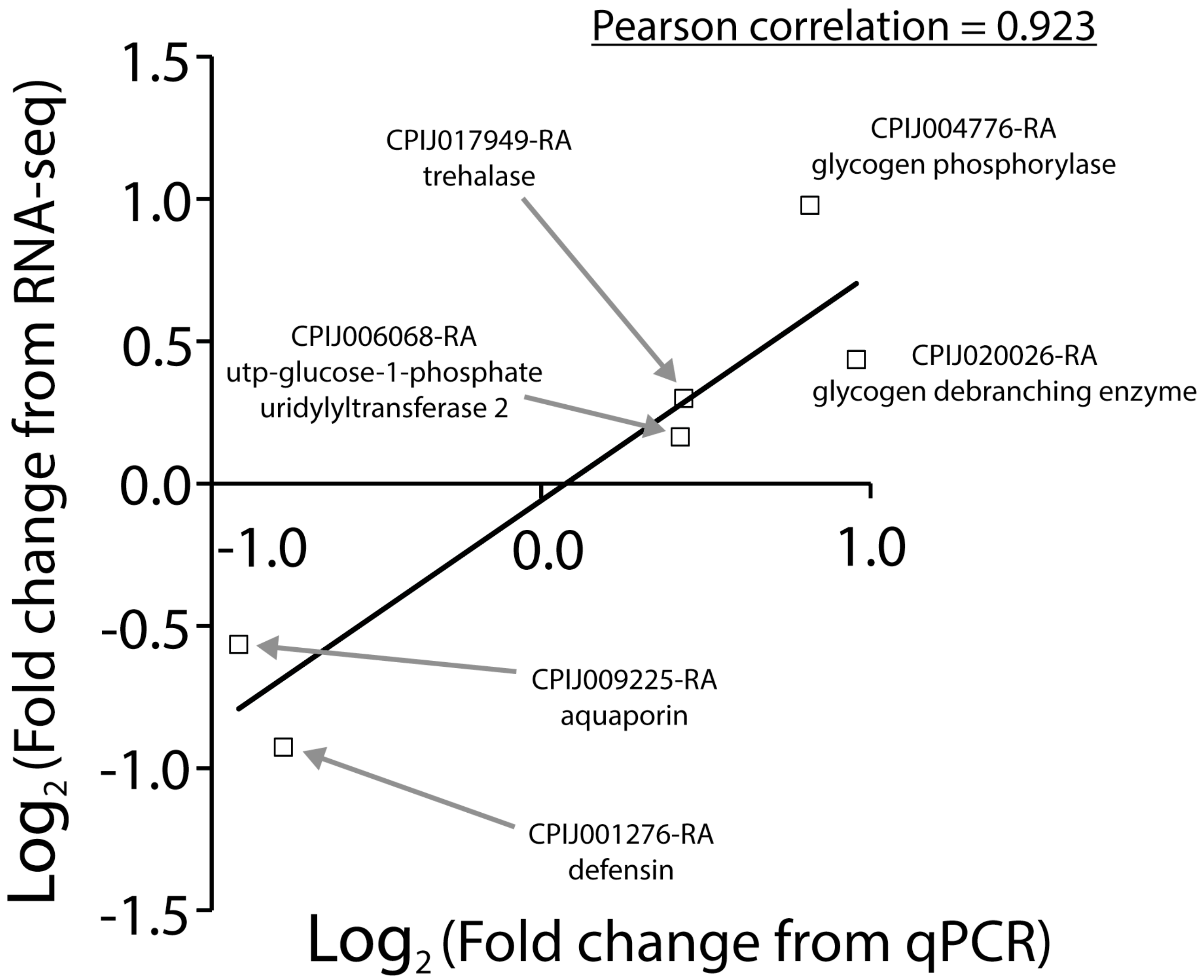

Figure S4: RNA-seq validation by qPCR. Each qPCR measurement was replicated four times. RNA-seq and qPCR results were compared with a Pearson's Correlation Coefficient.

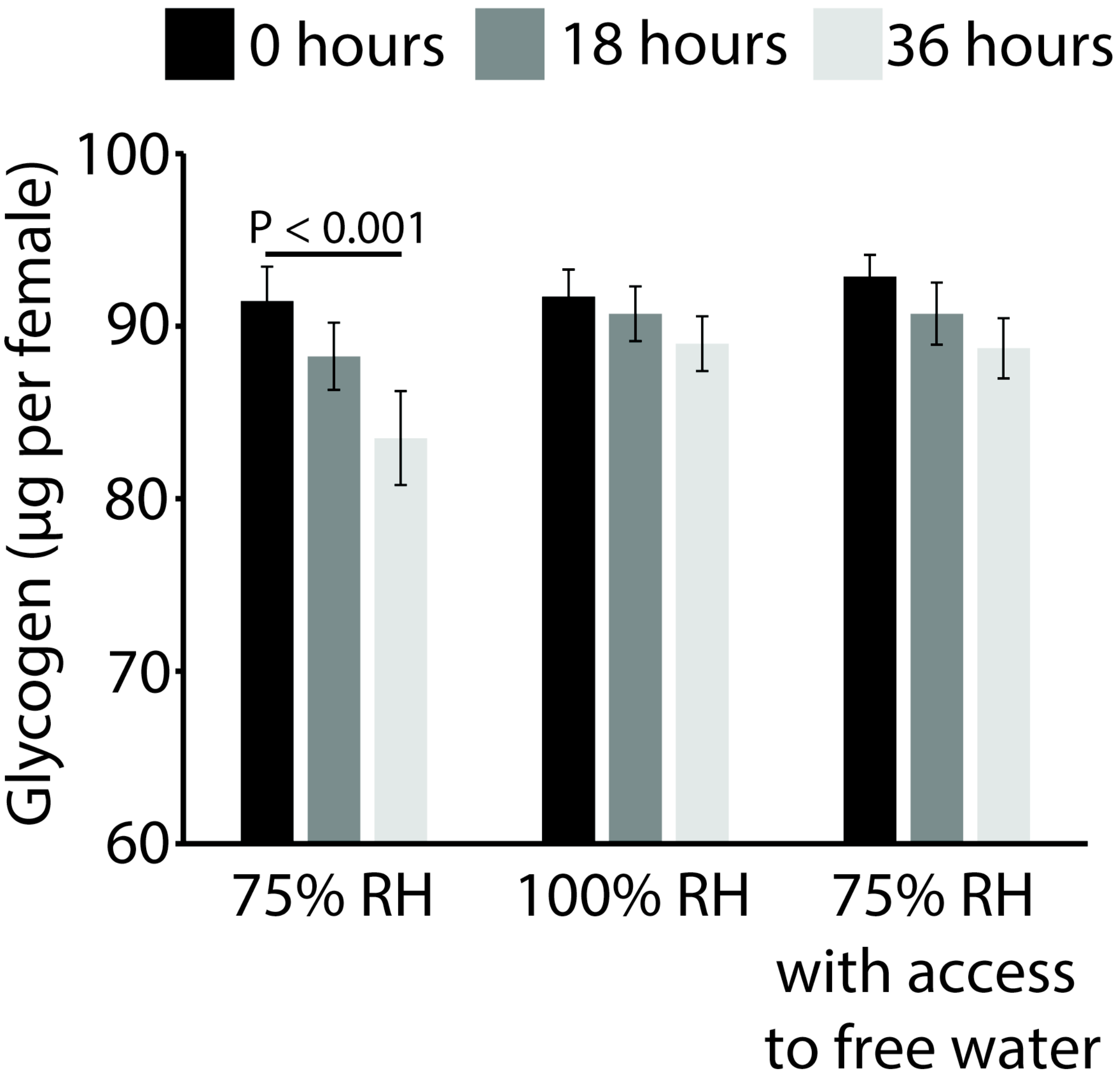

Figure S5: Glycogen content for *Culex pipiens* when held under 75% relative humidity (RH), 100% RH, and 75% RH with free access to water. Mean ± SE for 8 mosquitoes at each time point. Statistical analyses were conducted by a one- or two-way ANOVA followed by Tukey's HSD post-hoc analysis.

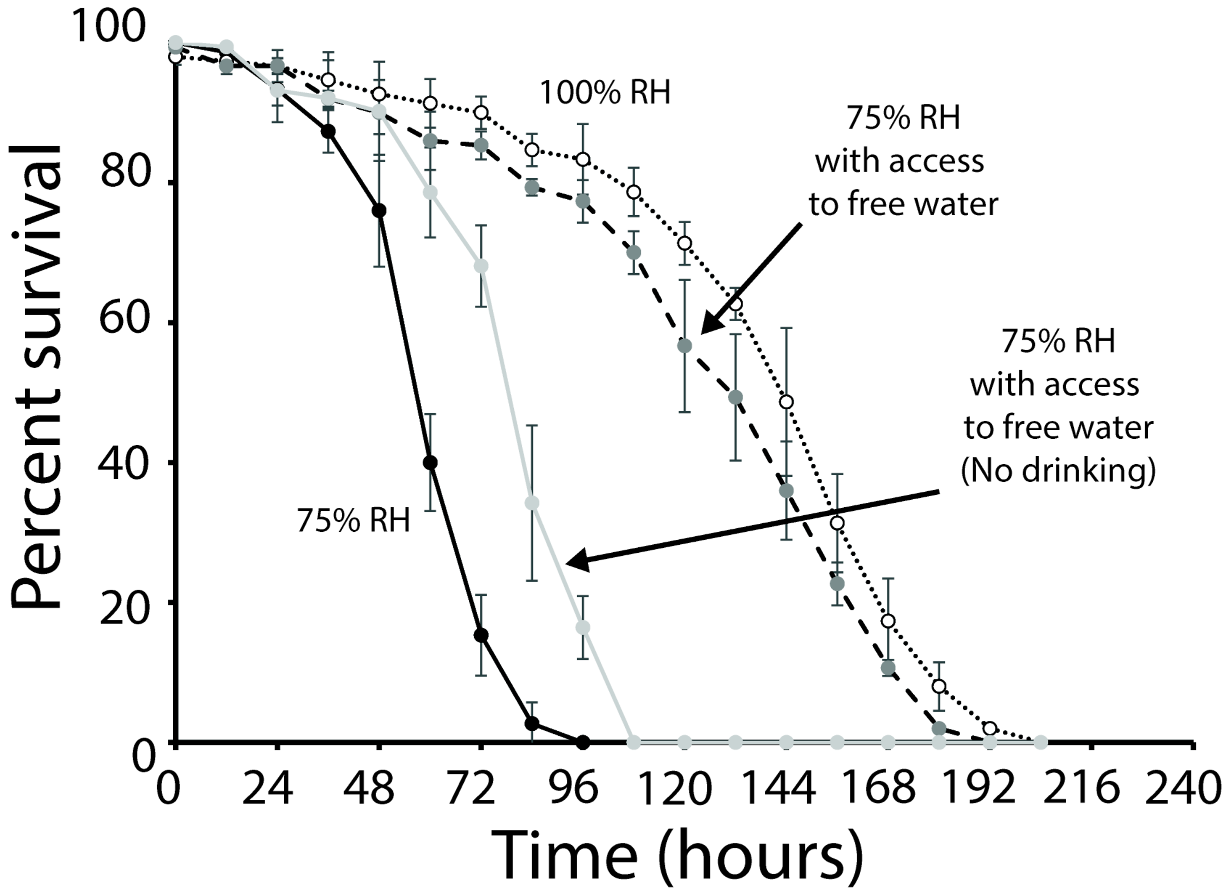

Figure S6: Survival of mosquitoes when held under 75% relative humidity (RH), 100% RH, 75% RH with free access to water, and 75% RH with free access to water (with no drinking). Mean ± SE for 3 replicates of 50 mosquitoes at each time point. Differences in survival were assessed through a probit analysis.

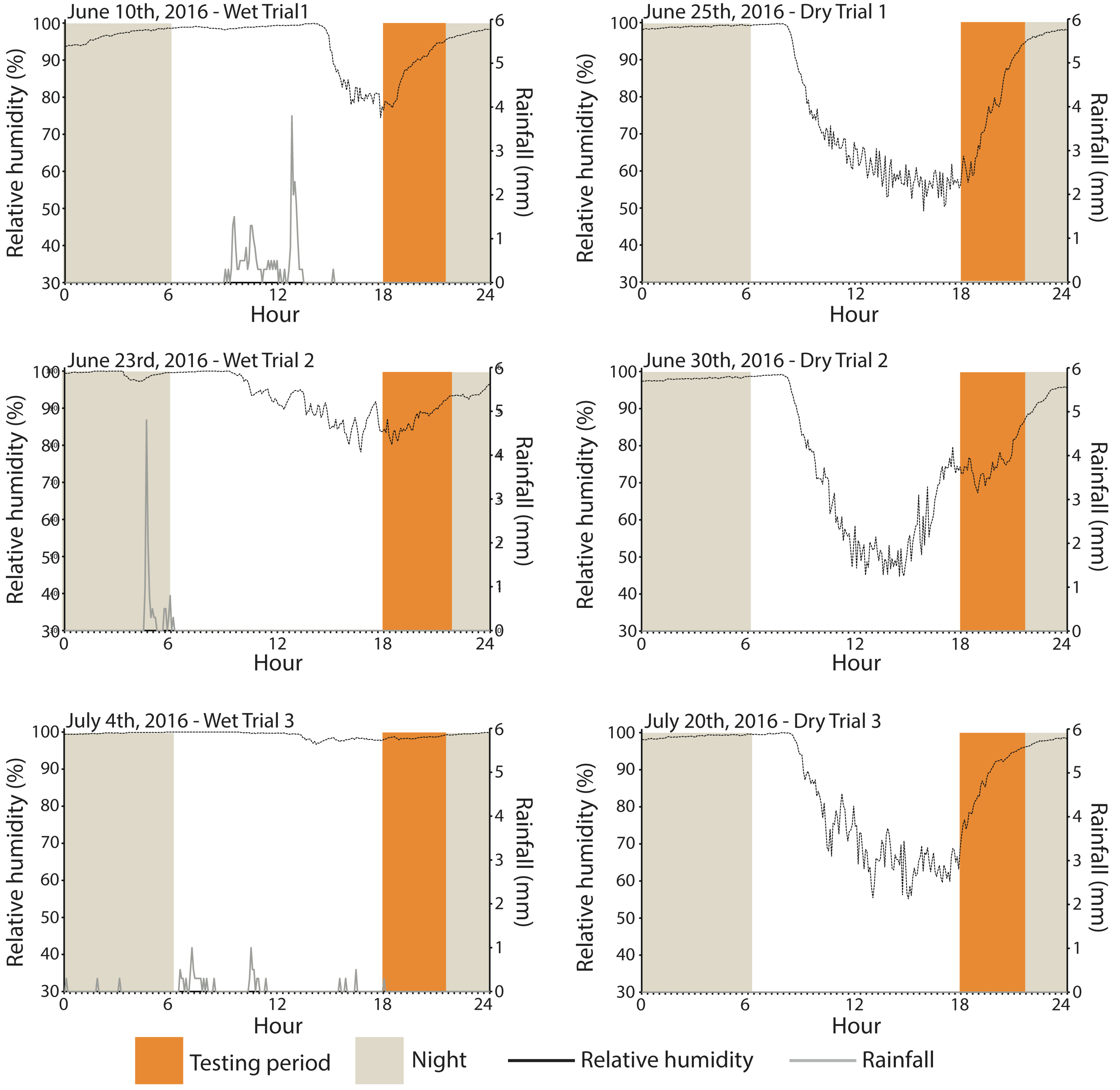

Figure S7: Relative humidity and rainfall during the 24 hour testing period for mesocosm studies. Left panels, wet trial periods. Right panels, dry trial periods. Gray areas, denotes dark periods. Orange areas, time of testing for landing on a host.

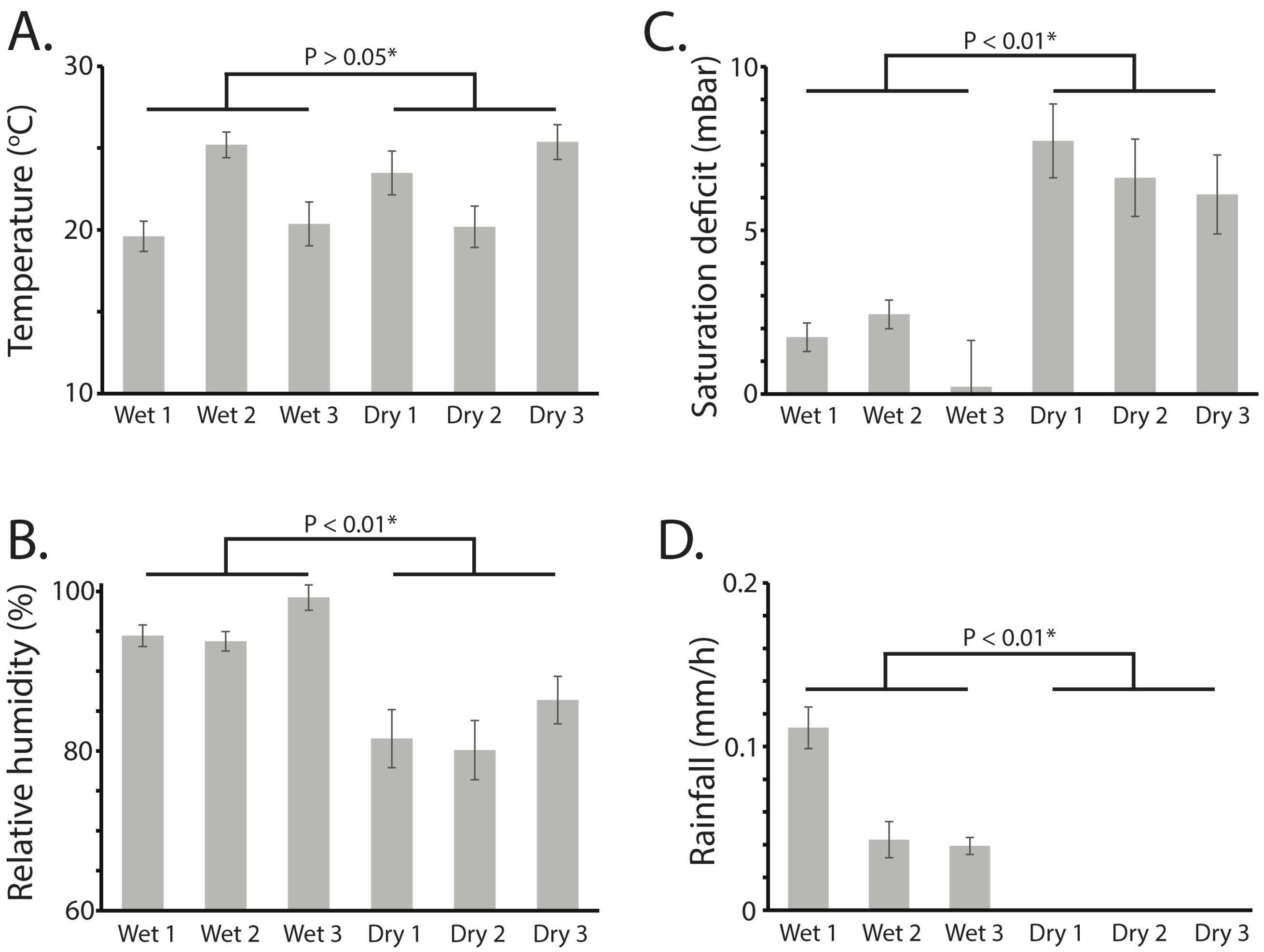

Figure S8: Environmental parameters during the 24 hour testing periods associated with mesocosm studies. A, temperature; B, relative humidity; C, saturation deficit; D, rainfall. Statistical analyses were conducted by ANOVA followed by Dunnett's post-hoc analysis. *, denotes differences between individual wet and dry groups.

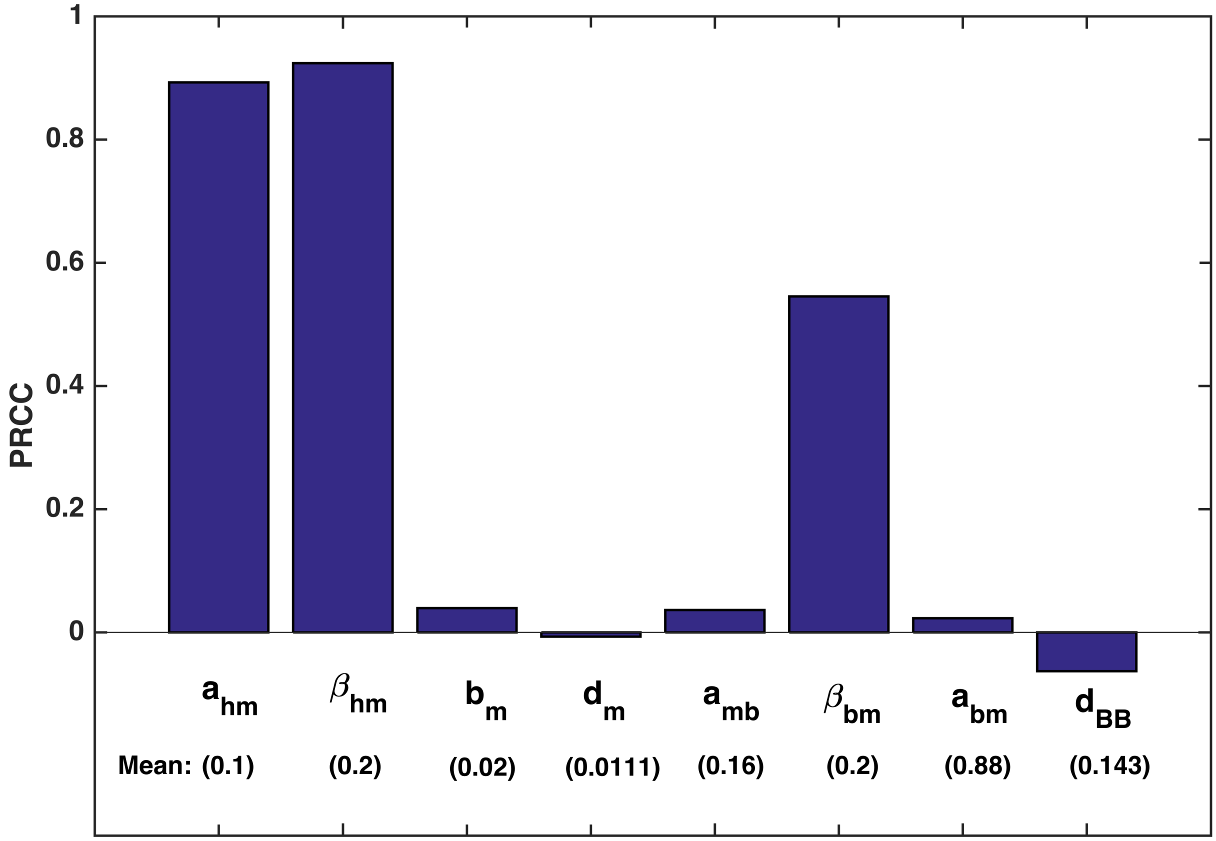

Figure S9: Sensitivity analysis of system (1) for the output variable, number of cumulated human infections, on some parameters: effective transmission rate from mosquito to human (a_{hm}), biting rates for humans (β_{hm}), mosquito birth ($b_{m)}$ and death (dm) rate, effective transmission rate from bird to mosquito (a_{mb}), biting rates for birs (β_{bm}), effective transmission rate from mosquito to bird (a_{bm}), and bird death (d_{bb}).

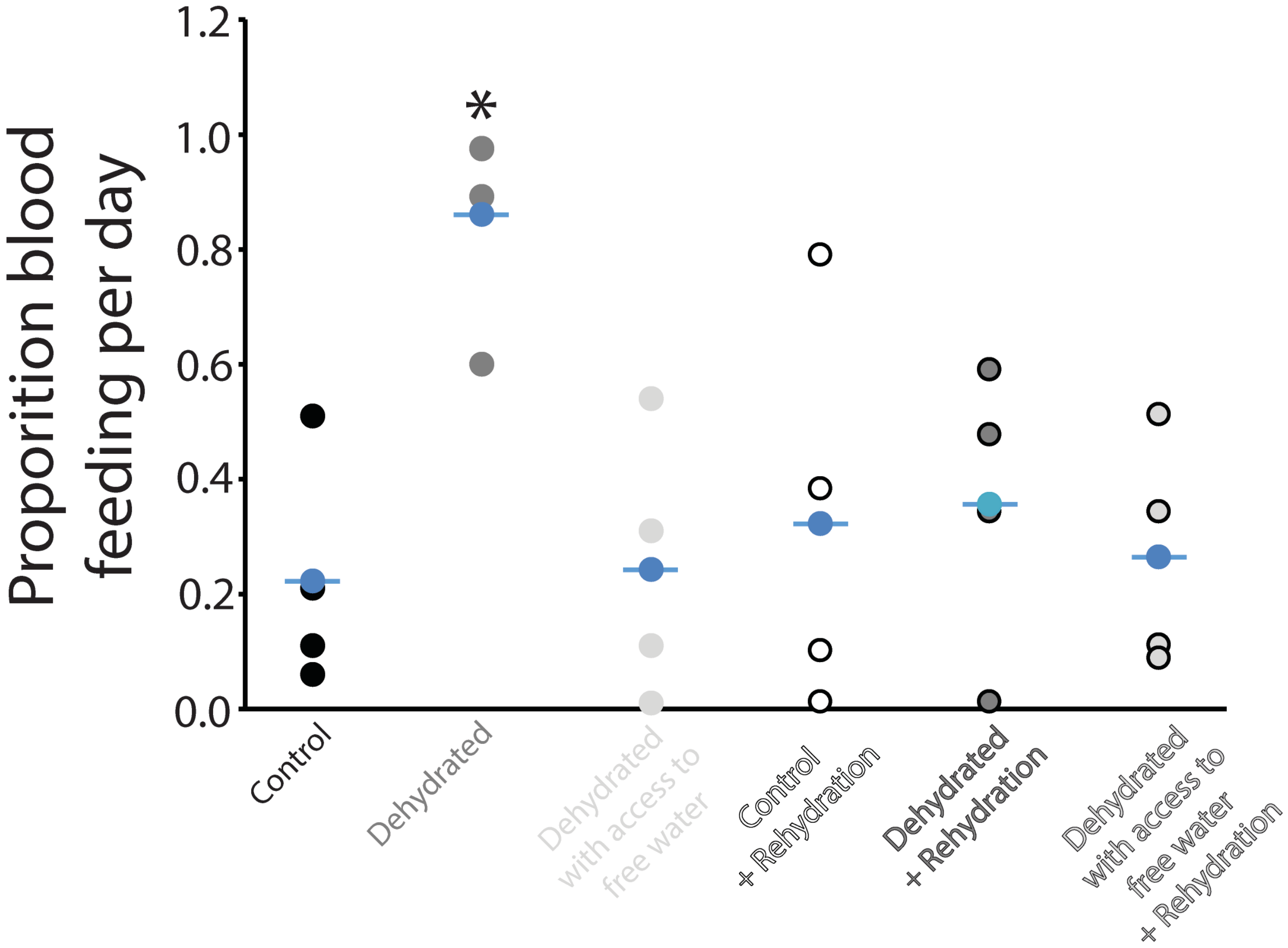

Figure S10: Proportion of mosquitoes (*Culex pipiens*) blood feeding held under different dehydration, rehydration, and dehydrating conditions with access to water protocols. Statistical analyses were conducted by ANOVA followed by Dunnett's post-hoc analysis. *, $P < 0.05$.

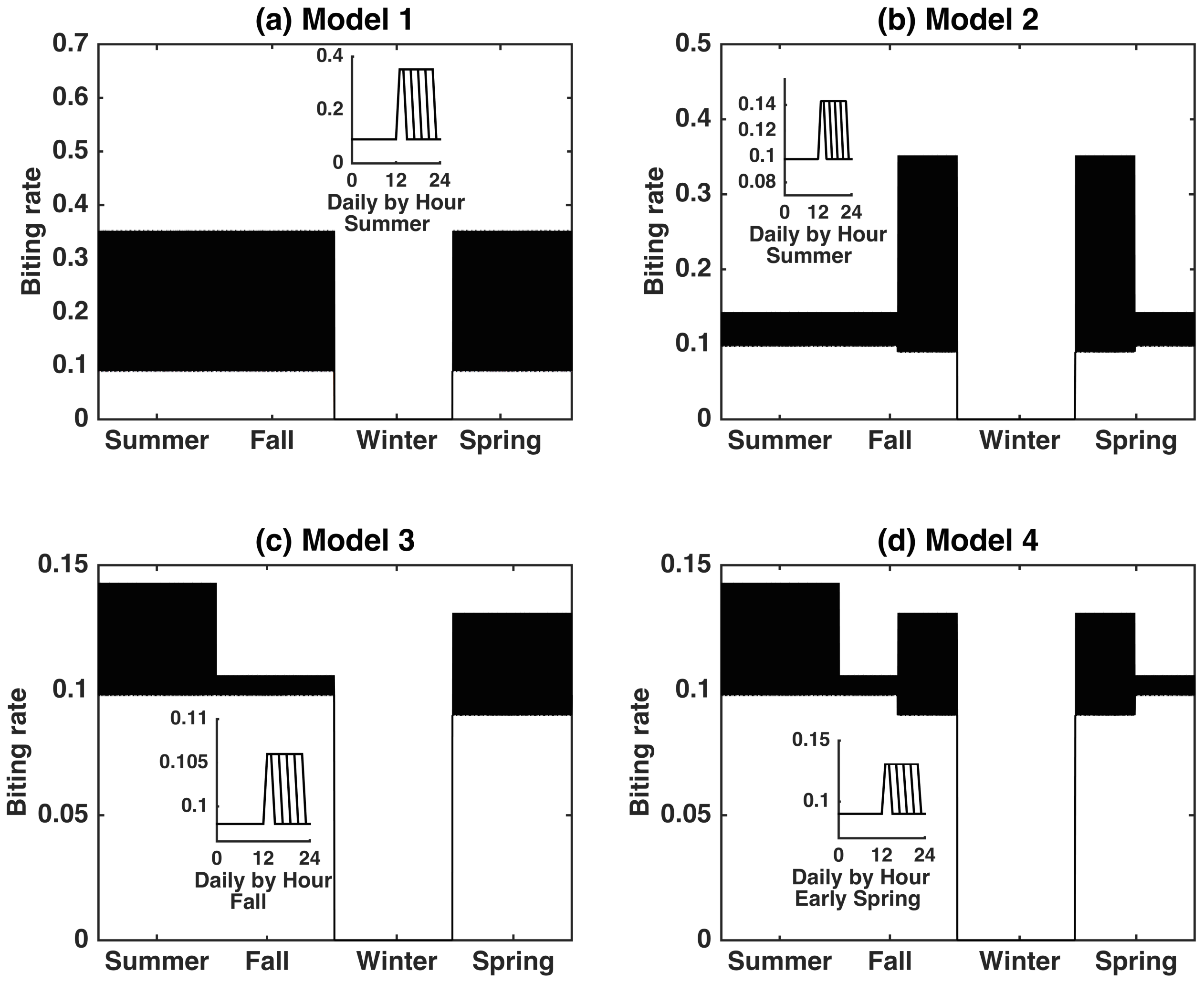

Figure S11: Biting rates for Models 1-4. Panel (a)- (d) show the functions for biting rates during different seasons and the subpanels inside each show the daily changes on biting rate due to multiple bouts of dehydration from 14:00 each day (2 hours – 10 hours).

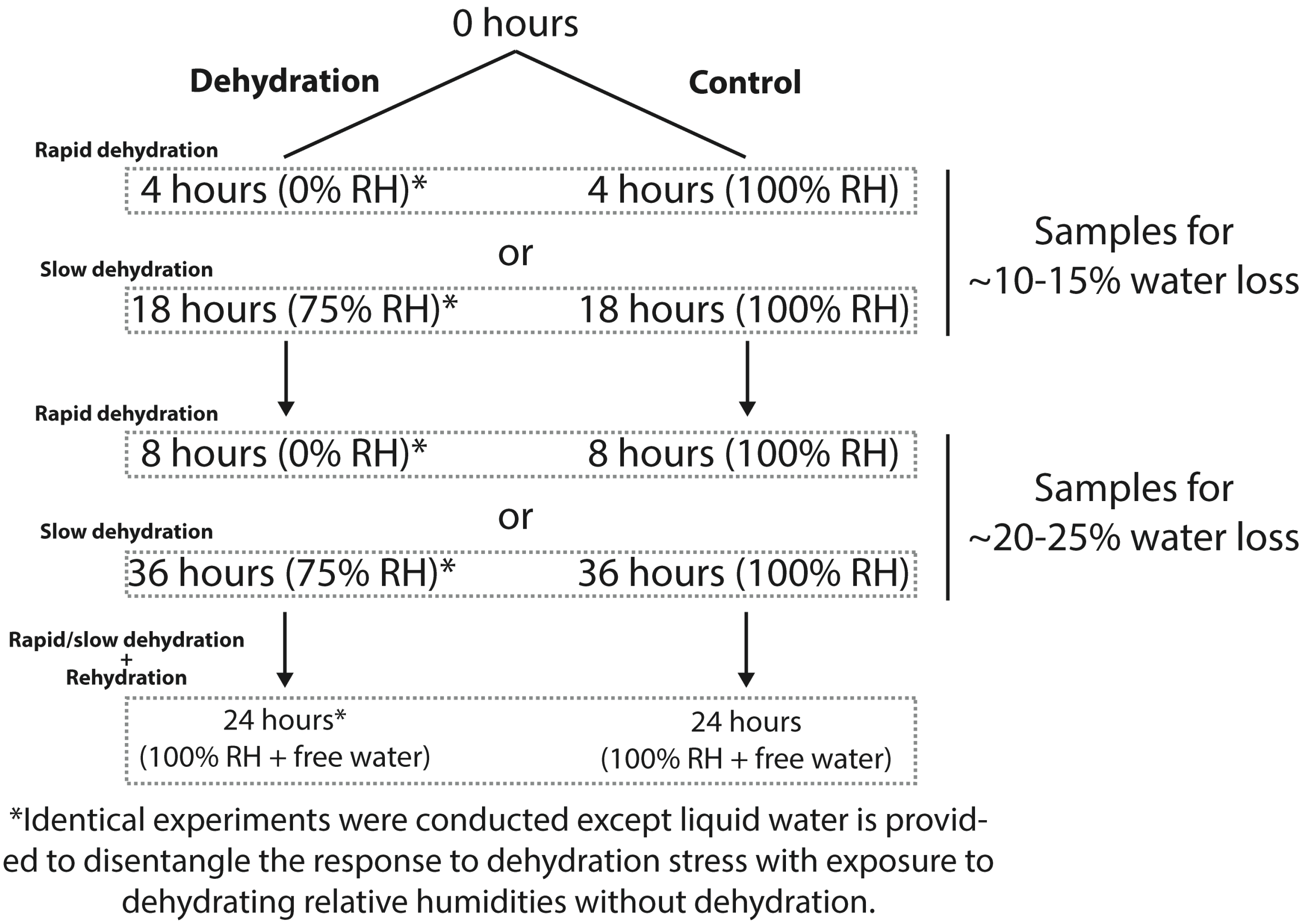

Figure S12: Schematic for dehydration exposures illustrating both dehydration and rehydration protocols. Each dehydration/rehydration point was not used for all assays.
